# The NIN-Like Protein OsNLP2 Negatively Regulates Ferroptotic Cell Death and Immune Responses to *Magnaporthe oryzae* in Rice

**DOI:** 10.3390/antiox11091795

**Published:** 2022-09-12

**Authors:** Yafei Chen, Juan Wang, Nam Khoa Nguyen, Byung Kook Hwang, Nam Soo Jwa

**Affiliations:** 1 Division of Integrative Bioscience and Biotechnology, College of Life Sciences, Sejong University, Seoul 05006, Korea; 2 State Key Laboratory of Agricultural Microbiology and Hubei Key Laboratory of Plant Pathology, College of Plant Science and Technology, Huazhong Agricultural University, Wuhan 430070, China; 3 Division of Biotechnology, College of Life Sciences and Biotechnology, Korea University, Seoul 06213, Korea

**Keywords:** cell death, ferroptosis, iron, *Magnaporthe oryzae*, nodule inception (NIN)-like protein, *Oryza sativa*, plant immunity, ROS

## Abstract

Nodule inception (NIN)-like proteins (NLPs) have a central role in nitrate signaling to mediate plant growth and development. Here, we report that OsNLP2 negatively regulates ferroptotic cell death and immune responses in rice during *Magnaporthe oryzae* infection. OsNLP2 was localized to the plant cell nucleus, suggesting that it acts as a transcription factor. OsNLP2 expression was involved in susceptible disease development. *ΔOsnlp2* knockout mutants exhibited reactive oxygen species (ROS) and iron-dependent ferroptotic hypersensitive response (HR) cell death in response to *M. oryzae*. Treatments with the iron chelator deferoxamine, lipid-ROS scavenger ferrostatin-1, actin polymerization inhibitor cytochalasin A, and NADPH oxidase inhibitor diphenyleneiodonium suppressed the accumulation of ROS and ferric ions, lipid peroxidation, and HR cell death, which ultimately led to successful *M. oryzae* colonization in *ΔOsnlp2* mutants. The loss-of-function of OsNLP2 triggered the expression of defense-related genes including *OsPBZ1*, *OsPIP-3A*, *OsWRKY104*, and *OsRbohB* in *ΔOsnlp2* mutants. *ΔOsnlp2* mutants exhibited broad-spectrum, nonspecific resistance to diverse *M. oryzae* strains. These combined results suggest that OsNLP2 acts as a negative regulator of ferroptotic HR cell death and defense responses in rice, and may be a valuable gene source for molecular breeding of rice with broad-spectrum resistance to blast disease.

## 1. Introduction

Plants have evolved a sophisticated immune system to overcome attacks by microbial pathogens [1,2]. Microbial pathogens have evolved pathogen effectors to promote virulence and cause disease in host plants [3]. The interaction between rice (*Oryza sativa* L.) and *Magnaporthe oryzae* is a useful model system to investigate the molecular and biochemical mechanisms underlying plant immunity and disease. Avirulent *M. oryzae* infection causes highly resistant and immune responses in rice cells, including defense-related gene expression, reactive oxygen species (ROS) bursts, and ferroptotic cell death [4,5,6,7]. ROS act as cellular signaling molecules to trigger plant immune responses [3]. ROS accumulation suppresses *M. oryzae* effector BAS4 activity to prevent *M. oryzae* infection [8].

Ferroptosis, a nonapoptotic form of iron-dependent cell death [9], is triggered by the accumulation of ferric ion (Fe^3+^) and toxic lipid ROS in mammalian cells [10]. Highly reactive Fe^2+^ reacts with H_2_O_2_ to produce Fe^3+^ and highly toxic ·OH (Fenton reaction) [11,12], which results in iron-dependent accumulation of toxic lipid ROS [9]. The hydroxyl radicals produced by the Fenton reaction causes severe damage to lipids, proteins, DNA, and cell components [13,14]. We recently reported that iron- and ROS-dependent signaling cascades are involved in the ferroptotic cell death pathway in rice during avirulent *M. oryzae* infection [4]. Rice MAP kinase (OsMEK2 and OsMPK1) signaling cascades are required for iron- and ROS-dependent ferroptotic cell death responses of rice to *M. oryzae* infection [5]. Avirulent *M. oryzae* infection in rice triggers the accumulation of Fe^3+^ and ROS accumulation and hypersensitive response (HR) cell death. ROS bursts induce HR cell death in rice in response to avirulent *M. oryzae* infection [15,16]. NADPH oxidases, also known as respiratory burst oxidase homologs (Rbohs), play a crucial role in ROS production in rice cells [17]. Cytoplasmic NADP-malic enzyme (ME) supplies electrons to plasma membrane-bound Rbohs for apoplastic ROS generation, leading to ferroptotic cell death in rice during *M. oryzae* infection [3,4,16]. By contrast, virulent *M. oryzae* infection in rice leaf sheath cells does not trigger iron and ROS accumulation and HR cell death [4]. In compatible rice–*M. oryzae* interactions, rice disease (susceptibility)-related genes may be involved in the suppression of iron- and ROS-dependent ferroptotic cell death responses during virulent *M. oryzae* infection.

Plants require nitrogen for growth, development, and defense against abiotic and biotic stresses [18]. Different nitrogen species differentially affect plant defense against invasive microbial pathogens. Nitrate (NO^3–^) nutrition enhances salicylic acid (SA), nitric oxide (NO), and HR cell death; by contrast, ammonium (NH_4_^+^) nutrition compromises disease resistance by enhancing the availability of nitrogen nutrients to pathogens [19]. Plant-specific RWP-RK proteins, such as the nodule inception (NIN)-like proteins NLP4 and NLP7, coordinate plant responses to nitrogen availability [20,21,22]. RWP-RK proteins contain a characteristic RWP-RK motif (a conserved amino acid sequence Arg-Trp-Pro-X-Arg-Lys, where X indicates any amino acid), which is responsible for DNA binding [21,23]. The RWP-RK proteins are classified into two subfamilies: the RWP-RK domain proteins (RKDs) and the NIN-like proteins (NLPs) with an additional Phox and Bem1 (PB1) domain at their C-terminus [21]. The PB1 domain functions in protein–protein interactions in plants [23].

The RWP-RKs family genes are expressed in almost all organs in *Arabidopsis* and rice during plant development and growth [21]. AtRKDs are involved in cell differentiation and normal gametophytic development [24]. The *AtRKD1* to *AtRKD4* genes are expressed primarily in reproductive organs [25]. The *Arabidopsis NLP7* gene regulates nitrate signaling in the presence of ammonium [26]. In rice, 16 RWP-RK proteins have been identified: ten belong to RKDs, and six are NLPs [21]. OsNLP1, a key regulator of nitrogen utilization, rapidly responds to nitrogen deficiency and enhances nitrogen use efficiency (NUE) and yield in rice [27]. OsNLP4 overexpression substantially increases rice yield and NUE under normal nitrogen levels [22]. Currently, it is not known whether rice NLP proteins are involved in disease, cell death, and immunity.

In this study, we isolated the rice NIN-like protein gene *OsNLP2* from a rice cDNA library [28]. OsNLP2 contained the RWP-RK domain and C-terminal PB1 domain. *OsNLP2* expression was induced in rice leaf sheaths during virulent *M. oryzae* infection but not during avirulent *M. oryzae* infection. OsNLP2 was localized to the nucleus, suggesting that it acts as a transcription factor that binds to a specific nucleotide sequence of target genes. We used T-DNA insertion *ΔOsnlp2* knockout mutants to investigate the role of OsNLP2 in ferroptotic cell death and defense responses to *M. oryzae* infection. A complementation test identified OsNLP2 as the causal gene for *ΔOsnlp2* mutant phenotypes. OsNLP2 knockout in rice triggered broad-spectrum, nonspecific resistance to different *M. oryzae* strains. Some defense-related genes in *ΔOsnlp2* mutants were highly expressed, such as *OsWRKY104* and *OsRbohB*, and abundant ROS and ferric ions (Fe^3+^) accumulated to induce HR ferroptotic cell death during *M. oryzae* infection. The results of this study indicate that OsNLP2 acts as a putative disease (susceptibility)-related gene to promote the infection and spread of diverse *M. oryzae* strains in rice cells. However, it is unclear how OsNLP2 suppresses plant cell death and immune responses to promote pathogen invasion. We showed that NLP proteins may negatively regulate HR cell death and defense responses to create a pathogen-compatible environment inside plant cells.

## 2. Materials and Methods

### 2.1. Plant Materials and Growth Conditions

Seeds of rice (*Oryza sativa* L.) cultivar Donjin (DJ) were provided by the National Institute of Crop Science, Jeonju, Korea (http://www.nics.go.kr, accessed on 1 September 2010). *ΔOsnlp2* knockout mutant seeds were obtained from the Rice Functional Genomic Express Database (RiceGE) managed by the Salk Institute (http://signal.salk.edu./cgi-bin/RiceGE, accessed on 8 March 2017) [29]. Rice seeds were germinated in water at 28 °C under continuous light (80 μmol photons m^−2^ s^−1^) for 5 days. Germinated rice seeds were planted in pots (12 cm diameter, 11 cm height) containing Baroker soil (Seoul Bio, Seoul, Korea). The plants were grown in a growth chamber under the following conditions: 28 °C, 60% humidity, white fluorescent light (150 µmol photons m^−2^ s^−1^), and a 16 h day/8 h night photoperiod.

*Nicotiana benthamiana* seeds were germinated in water for 7 days under continuous light at 25 °C. Germinated seedlings were planted in pots (8 cm diameter, 6 cm height) containing Baroker soil (Seoul Bio, Seoul, Korea). *N. benthamiana* plants were grown in a growth chamber under the following conditions: 25 °C, white fluorescent light (150 µmol photons m^−2^ s^−1^), and a 16 h day/8 h night photoperiod.

### 2.2. ΔOsnlp2 Mutant Genotyping

T-DNA insertion *ΔOsnlp2* mutant seeds were sterilized as follows: seed husks were removed, and seeds were treated with 100% ethanol for 1 min and 50% Clorox for 30 min. Sterilized seeds were cultured in one-half Murashige and Skoog (MS) (Sigma-Aldrich, St. Louis, MO, USA) medium at 25 °C under continuous light for 2 weeks. Fresh rice leaves were collected from each plant to extract genomic DNA for genotyping. The cetyltrimethylammonium bromide (CTAB) method was used to extract genomic DNA [30]. The primers were designed near the T-DNA insertion site based on information from the mutant database (http://signal.salk.edu./cgi-bin/RiceGE, accessed on 8 March 2017) to detect homozygous and heterozygous plants from the seed pool. The gene-specific primers and T-DNA right-border primer (RB) were used to verify the T-DNA insertion in *ΔOsnlp2* mutant plants. The primers were in Table S1. To germinate *ΔOsnlp2* mutant seeds to grow to the whole plants, the peeled seeds of *ΔOsnlp2* mutants were cultured in one-half MS media containing 30 mg·L^−1^ hygromycin.

Total RNA was extracted from homozygous plants using TRIzol reagent (Invitrogen, Carlsbad, CA, USA) and then used to synthesize cDNAs. *OsNLP2* transcriptional levels in rice DJ and *ΔOsnlp2* mutant lines were analyzed by performing RT-PCR and a real-time quantitative reverse transcription polymerase chain reaction (real-time qRT-PCR) using *OsNLP2* RT primers. Rice *Ubiquitin* (*OsUbi*) transcript levels were used to normalize the transcript levels of *OsNLP2*. The data are presented as means ± SD of relative expression quantities of *OsNLP2* in leaf sheaths from different rice plants (*n* = 4).

### 2.3. Fungal Cultures and Growth Conditions

*M. oryzae* strains PO6-6, KJ401, Y34, RO1-1, 70-15, and 007 were obtained from the Center for Fungal Genetic Resources, Seoul National University, Seoul, Korea (http://genebank.snu.ac.kr, accessed on 5 January 2010). *M. oryzae* PO6-6, KJ401, Y34, RO1-1, and 70-15 are virulent to rice DJ, whereas *M. oryzae* 007 is avirulent to rice DJ. *M. oryzae* strains were stored at −20 °C and cultured on rice bran agar media (20 g rice bran, 20 g sucrose, and 20 g agar in 1 L water) in the dark at 25 °C for 14 days [4,5]. Sporulation of *M. oryzae* cultures was induced by incubating the culture plates under continuous fluorescent light (80 µmol photons m^−2^ s^−1^) for 3 days. Conidial suspensions in 0.025% (*v*/*v*) Tween 20 were adjusted to appropriate conidial concentrations.

### 2.4. Fungal Inoculation and Infection Evaluation

To monitor disease symptoms on rice leaves, *M. oryzae* conidial suspensions (1.0 × 10^5^ conidia mL^−1^) were uniformly sprayed on leaves of 3-week-old seedling plants. Inoculated rice plants were incubated at 25 °C under dark and moist conditions for 24 h, and then placed under normal conditions (16 h day/8 h night). Disease symptoms on rice leaves were monitored 5 days after inoculation.

For the rice leaf sheath tests, *M. oryzae* conidial suspensions (5.0 × 10^5^ conidia mL^−1^) were inoculated on 6-week-old rice leaf sheaths (5 cm length). Inoculated rice sheaths were incubated in the dark for 2 days in a moistened chamber. Then, epidermal 3–4 cell thick layers were excised from the rice sheaths and monitored under a fluorescence microscope (Zeiss equipped with Axioplan 2; ZEISS, Campbell, CA, USA) as described previously [4]. Infected epidermal cells were categorized into three infection types: expanded infection (invasive hyphae colonized inside multiple cells), single-cell infection (limited hyphal growth inside a single cell), and HR cell death. Infected cells of each infection type were counted using four replicates from each of three independent experiments.

### 2.5. Cloning and Plasmid Construction

To detect the subcellular localization of OsNLP2 and its functional domains, entry clones were recombined into the Gateway green fluorescent protein (GFP)-containing vector pGWB552 using the Gateway LR Clonase II enzyme (Invitrogen, Carlsbad, CA, USA) [31]. The coding sequences of *OsNLP2* (LOC_Os04g41850) and its functional domains were amplified from the rice cDNA library using gene-specific primers containing Gateway attB1 and attB2 sites. The gene-specific primers were designed based on genome information provided by the Rice Genome Annotation Project (http://rice.plantbiology.msu.edu/, accessed on 5 June 2016). The primers were list in Table S1.

For the transient *OsNLP2* expression assay in *N. benthamiana* leaves, the OsNLP2 coding region and its functional domains were cloned into the ligation-independent cloning (LIC) vector pCAM2300-LIC. These coding sequences were amplified from the rice cDNA library using primers with adaptors. Amplified PCR products were subcloned into the pCAM2300-LIC vector digested with a *SnaB1* restriction enzyme using T4 DNA polymerase (New England Biolabs, Ipswich, MA, USA).

### 2.6. Agrobacterium Transformation, OsNLP2 Subcellular Localization, and Transient OsNLP2 Expression Assay

To determine the subcellular localization of OsNLP2, *OsNLP2* full sequence and functional domains in pGWB552:GFP were transformed into *Agrobacterium* GV3101. The empty pGWB552:GFP vector was transformed as a negative control. *Agrobacteria* transformed with different constructs were cultured overnight at 28 °C in Luria Bertani (LB) liquid medium containing 100 µg mL^−1^ spectinomycin. The *Agrobacterium* cells were collected and then suspended in an infiltration buffer [97.5 mg 2-(*N*-morpholino)ethanesulfonic acid sodium salt (MES), 0.5 mL of 1 M MgCl_2_, and 0.1 mM acetosyringone in 50 mL water]. The *Agrobacterium* suspensions were infiltrated into *N. benthamiana* leaves, incubated for 2 days, and then the infiltrated leaves were stained with 4′,6-diamidino-2-phenylindole (DAPI) solution (1 µg mL^−1^) for 10 min in the dark to visualize nuclei. The epidermal cells were observed using a fluorescence microscope (Zeiss equipped with Axioplan 2) with green fluorescence filters (Ex/Em: 488 nm/505–550 nm wavelengths) and DAPI filters (Ex/Em: 405/421–523 nm).

To transiently express OsNLP2 in *N. benthamiana* leaves, full-length *OsNLP2* and its functional domains in the binary vector pCAM2300-LIC were transformed into *Agrobacterium* GV3101. The *Agrobacterium* suspensions in the infiltration buffer were adjusted to a final concentration of OD_600_ = 1.0. The transformed *Agrobacterium* suspensions containing each of the three different constructs were infiltrated into individual sites on the same *N. benthamiana* leaves. The empty pCAM2300-LIC vector and Infestin 1 (INF1):pCAM2300-LIC were used as negative and positive controls, respectively. Cell death symptoms were observed on infiltrated *N. benthamiana* leaves after incubating for 2 days.

### 2.7. Complementation of OsNLP2 into ΔOsnlp2 Mutant

To restore the expression of *OsNLP2* in *Δosnlp2* mutant plants, the *OsNLP2* coding sequence inside the binary vector pB2GW7 (*Bar* gene as the selection marker) was transformed into *Agrobacterium C58C1* [32]. The *OsNLP2-*complemented plants were created using the *ΔOsnlp2* mutant line #6-4. Briefly, 4-week-old rice calli of *ΔOsnlp2* mutant line #6-4 were used for the 3-day co-culture with *Agrobacterium C58C1* containing OsNLP2:pB2GW7. The transformed calli were selected on the media containing increasing concentrations of phosphinothricin (PPT, 3 mg/L and 6 mg/L). After shooting and rooting, the calli were cultured in the regeneration media for 2 weeks in light at 25 °C, and transferred to soil after 2-day adaption in water. *OsNLP2* primers and *Bar* gene primers were used to confirm the *OsNLP2* expression in the transformants (Table S1).

### 2.8. RT-PCR and Real-Time qRT-PCR Analyses

Rice gene expression levels in *M. oryzae*-infected leaf sheath tissues were analyzed by reverse-transcription PCR (RT-PCR) and real-time quantitative PCR (qRT-PCR). Total RNAs were extracted from rice tissues using TRIzol reagent (Invitrogen), followed by first-strand cDNA syntheses using a cDNA synthesis kit (Invitrogen) according to the manufacturer’s instructions. Equal amounts of cDNAs were used as templates for RT-PCR and real-time qRT-PCR. The expression of *OsNLP2*, *OsPBZ1*, *OsWRKY104*, *OsRbohB*, *OsPIP-3A*, and *OsWRKY90* was analyzed with gene-specific primer sets by real-time qRT-PCR using TOPreal qPCR 2× PreMIX (SYBR Green with low ROX, Enzynomics). Relative expression levels of the tested rice genes were determined by normalizing them with respect to the expression levels of rice *OsUbiquitin, 18S rRNA*, and *OsActin* as internal controls. The relative expression values were calculated using the ΔΔCt method [33]. The data are means ± SD of relative gene expression levels in leaf sheaths from three independent experiments. The list of gene-specific primers used in this study is provided in Table S1.

### 2.9. ROS Detection and Quantification

Cellular ROS (H_2_O_2_) localization in rice leaf sheaths infected with *M. oryzae* was visualized using the following dyes: 5-(and 6-)chloromethyl-2′,7′-dichlorodihydrofluorescein diacetate acetyl ester (CM-H_2_DCFDA) and 3,3′-diaminobenzidine (DAB). CM-H_2_DCFDA reacts with ROS in living cells and produces 2′,7′-dichlorofluorescein (DCF), a highly fluorescent product that can be detected using a fluorescence microscope with GF filters [4,34]. The isolated epidermal layers of rice leaf sheaths were soaked in water for 5 min at 4 °C to wash away wound-induced ROS, followed by staining in 2 µM CM-H_2_DCFDA (Molecular Probes Life Technologies, Eugene, OH) for 30 min in the dark at room temperature. Samples were washed three times with 1× phosphate-buffered saline (PBS) buffer, and stained epidermal sheath cells were observed under a fluorescence microscope (Zeiss equipped with Axioplan 2) with green fluorescence (GF) filters (Ex/Em: 488 nm/505–550 nm wavelengths). The isolated epidermal layers from infected rice sheaths were incubated in 1 mg mL^−1^ DAB solution (Sigma-Aldrich, St. Louis, MO, USA) for 8 h at room temperature, and then destained with ethanol:acetic acid:glycerol (3:1:1, *v*/*v*/*v*) as described previously [4,34,35]. ROS localization inside epidermal cells was observed under a microscope.

ROS production in rice sheath cells after inoculation with *M. oryzae* was measured using the chemiluminescence assay [4,16]. The isolated epidermal layers were cut into small pieces (0.5 cm) and submerged in Milli-Q water for 5 min at 4 °C to reduce wound-induced ROS. A piece of the epidermal layer was transferred into each well of a 96-well plate containing a chemiluminescence solution [30 µL luminol (Bio-Rad, Hercules, CA, USA), 1 µL horseradish peroxidase (1 mg mL^−1^; Jackson Immunoresearch, West Grove, PA), and 69 µL Milli-Q water], and incubated in the dark for 5 min at room temperature. Chemiluminescence [relative luminescent units (RLU)] of the ROS signals was detected using a GloMax 96 Microplate Luminometer (Promega, Madison, WI, USA).

### 2.10. Prussian Blue Staining for Ferric Ion Detection

Ferric ion (Fe^3+^) accumulation in rice leaf sheath cells was visualized by performing Prussian blue staining [5,36]. Epidermal layers of rice sheaths were isolated and stained with a Prussian blue solution (7% potassium ferrocyanide and 2% hydrochloric acid) for 15 h [37]. The stained epidermal cells were observed under a microscope (Zeiss equipped with Axioplan 2). Ferric ferrocyanides, which combine with Fe^3+^ inside cells, appear as a bright blue color in sheath epidermal cells.

### 2.11. Lipid Peroxidation Assay

To detect lipid peroxidation levels in rice sheaths infected with *M. oryzae*, we quantified malondialdehyde (MDA), a product of unsaturated fatty acid peroxidation, by reacting it with thiobarbituric acid (TBA) and measuring with a spectrophotometer [4,38]. Rice leaf sheaths were ground with liquid nitrogen, and an equal amount of the powdered tissue was mixed with the TBA solution [0.5% (*w*/*v*) TBA, 20% (*v*/*v*) trichloroacetic acid (TCA), and 0.25 mL 175 mM NaCl in a total of 2 mL of 50 mM Tris-Cl pH 8.0]. The sample was incubated in boiling water for 5 min and centrifuged at 14,000× *g* for 5 min at 4 °C. The resulting supernatants were used to measure absorbance [optical density (OD)] with an SP-2000UV spectrophotometer (Woongki Science, Seoul, Korea) at 450, 532, and 600 nm wavelengths. MDA concentrations (C) were calculated according to the following equation: C_(MDA)_ = 6.45 × (OD_532_ − OD_600_) − (0.56 × OD_450_) [38].

### 2.12. Treatment with Deferoxamine, Ferrostatin-1, Cytochalasin A, and Diphenyleneiodonium

The iron chelator deferoxamine (DFO) [36], potent ferroptosis inhibitor ferrostatin-1 (Fer-1) [4,5,6,7,8,9], actin filament inhibitor cytochalasin A (Cyt A) [36], and oxidase inhibitor diphenyleneiodonium (DPI) [39] were used to investigate iron- and ROS-dependent ferroptotic cell death in leaf sheaths of *ΔOsnlp2* mutant and complementation plants during *M. oryzae* infection. DFO, Fer-1, and DPI were purchased from Sigma-Aldrich, and Cyt A was purchased from Cayman Chemical Company (Ann Arbor, MI, USA). For the iron chelator assay, the epidermal layer of rice leaf sheaths was isolated at 42 h post-inoculation (hpi) and incubated in 3 mM DFO for 6 h at room temperature. For Fer-1 treatment, the epidermal layer of rice leaf sheaths was isolated at 24 hpi and incubated in 10 µM Fer-1 for 24 h in the dark at room temperature after vacuum infiltration. For Cyt A and DPI treatments, the rice leaf sheaths (5–7 cm length) were inoculated with conidial suspension (5 × 10^5^ conidia mL^−1^) in water (mock), Cyt A (20 µg mL^−1^), or DPI (5 µM). The inoculated and treated rice leaf sheaths were then incubated in the dark at 25 °C for 48 h. The middle thin epidermal layers of the infected and treated rice leaf sheaths were stained and observed under a fluorescence microscope (Zeiss equipped with Axioplan 2).

## 3. Results

### 3.1. Identification of OsNLP2, ΔOsnlp2 Mutant, and OsNLP2 Complementation Plants

A truncated RWP-RK domain-containing protein was screened from the rice cDNA library and identified as a NIN-like protein (named OsNLP2) by BLAST (Basic Local Alignment Search Tool) analysis of a DNA sequence in the National Center of Biotechnology Information (NCBI) (https://www.ncbi.nlm.nih.gov/, accessed on 20 April 2016). NIN family proteins were identified as transcription factors (TFs) in nodulation and nitrate signaling [20,21]. We aligned the amino acid sequences of rice RWP-RK family proteins (NLP subfamily and RKD subfamily proteins) with other plant NLP proteins (*Arabidopsis thaliana*, *Medicago truncatula*, and *Brachypodium distachyon*) (Figure S1). The OsNLP2 used in this study had the GAF-like domain, RWP-RK domain (N567 to V615), and C-terminal PB1 domain (L835 to D915) (Figure S1). The GAF-like domain was detected at the N-terminal region of NLP subfamily proteins [21]. The function of the GAF-like domain in NLP proteins is not yet known. The PB1 domain contains a positively charged lysine (K) and a negatively charged OPCA motif (D-x-D/E-x-D/E) (Figure S1) [40]. Most of the aligned NLP proteins shared the conserved RWP-RK domain and PB1 domain, except for OsNLP6 that lacked the standard RWP-RK motif. OsRKD proteins lacked the PB1 domain. We constructed a phylogenetic tree using the neighbor-joining method and MEGA7 software (Version 7.0, created by Sudhir Kumar, Glen Stecher, and Koichiro Tamura, Kent, UK) (Figure S2). NLP subfamily proteins were distinctly separated from RKD subfamily proteins in the phylogenetic tree. The OsNLP2 used in this study was phylogenetically close to BdNLP4, OsNLP5, and BdNLP3 (Figure S2). The amino acid sequences of plant RWP-RK family proteins were aligned based on information from the Rice Genome Annotation Project (http://rice.plantbiology.msu.edu/, accessed on 2 June 2016), *Arabidopsis* Information Resource (TAIR) (http://www.arabidopsis.org/, accessed on 2 June 2016), Phytozome (http://www.phytozome.net/, accessed on 2 June 2016), and NCBI (https://www.ncbi.nlm.nih.gov/, accessed on 2 June 2016). Accession numbers of the aligned plant RWP-RK proteins are listed in Table S2.

### 3.2. OsNLP2 Expression Patterns in Rice–M. oryzae Interactions

**Figure 1:**
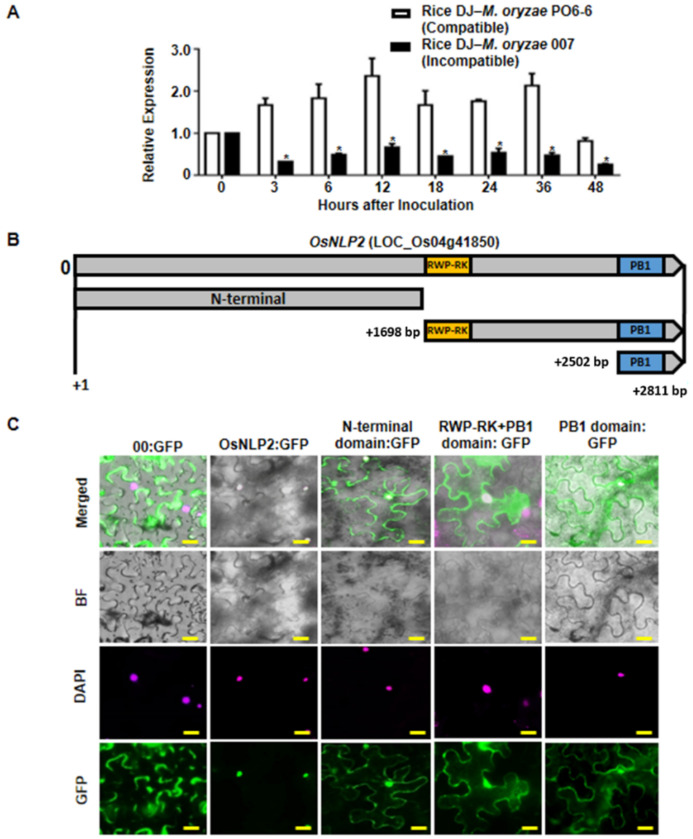
OsNLP2 expression pattern and subcellular localization analysis. (**A**). Real-time qRT-PCR analyses of *OsNLP2* expression in leaf sheaths of rice DJ at different time points after inoculation with *Magnaporthe oryzae* PO6-6 (virulent) and 007 (avirulent) strains. Transcript levels of the internal control genes *OsUbiquitin*, *18S rRNA*, and *OsActin* were used to normalize *OsNLP2* transcript levels. Relative expression levels were determined by comparing the values at different time points with the 0 h sample values. The data are presented as means ± standard deviation (SD) of relative expression quantities of *OsNLP2* in leaf sheaths from the different rice plants (*n =* 4). Asterisks indicate significant differences, as determined by Student’s *t*-test (*p* < 0.05). (**B**). Domain alignment of *OsNLP2*. The *OsNLP2* coding region is 2,811 bp. *OsNLP2* contains two functional domains (RWP-RK and PB1). Sequences and putative domain alignment were analyzed based on gene information from the rice genome annotation project (http://rice.plantbiology.msu.edu/, accessed on 5 June 2016) and the National Center of Biotechnology Information (NCBI, https://www.ncbi.nlm.nih.gov/, accessed on 5 June 2016). (**C**). Subcellular localization of OsNLP2:GFP and its truncated regions with either of the functional domains in *N. benthamiana* cells. DAPI counterstaining was performed to visualize nuclei. GFP and DAPI fluorescence were observed under a fluorescence microscope (Zeiss equipped with Axioplan 2) with a GF filter (Ex/Em: 488 nm/505–550 nm wavelengths) and DAPI filter (Ex/Em: 405/421–523 nm). ‘Merged’ shows the merged images of BF, GF, and DAPI. BF, bright field; GF, green fluorescence; GFP, green fluorescent protein; hpi, hours post infiltration. Scale bars = 10 μm.

To investigate whether *OsNLP2* expression is involved in rice disease and immunity, we analyzed the *OsNLP2* transcription levels in leaf sheaths of DJ rice during *M. oryzae* infection. Infected leaf sheaths of DJ (used as wild-type rice) were sampled at early time points after inoculation with *M. oryzae* PO6-6 (virulent) and 007 (avirulent) strains. Transcript levels of the internal control genes *OsUbiquitin* were used to normalize *OsNLP2* transcript levels (Figure 1A). Invariant expression of these reference genes normalized expression levels of *OsNLP2* in rice leaf sheaths during infection. Real-time qRT-PCR data showed that infection with virulent *M. oryzae* PO6-6 distinctly induced *OsNLP2* expression in DJ leaf sheaths at early infection stages (3–48 hpi). By contrast, infection with avirulent *M. oryzae* 007 did not induce *OsNLP2* expression by 48 hpi during incompatible rice–*M. oryzae* interactions. These expression patterns (Figure 1A) indicate that *OsNLP2* is involved in blast disease (susceptibility), and may act as a negative regulator to suppress rice HR cell death and defense responses to *M. oryzae* infection.

### 3.3. Subcellular Localization of OsNLP2

The plant-specific RWP-RK family of TFs are, at least partly, localized in the nucleus [21]. OsNLP2 contained the characteristic RWP-RK domain and C-terminal PB1 domain (Figure 1B and Figure S1), which may function in DNA-binding and protein–protein interactions, respectively [40]. The *OsNLP2* coding sequence and its N-terminal domain and RWP-RK or PB1 domain regions were constructed with a GFP tag, transiently expressed in *N. benthamiana* leaves using the agroinfiltration method; leaf cell nuclei were counterstained with DAPI; and cells were observed using a fluorescence microscope (Figure 1C). The control GFP construct (00:GFP) was ubiquitous in the plasma membrane and cytoplasm of *N. benthamiana* cells. Full-sequence OsNLP2:GFPs were localized only in the nuclei, overlapping with the DAPI fluorescence (Figure 1C). By contrast, the N-terminal domain:GFP was localized to both the plasma membrane and nuclei. RWP-RK + PB1:GFP and PB1:GFP were localized to both the cytoplasm and nuclei. This indicates that nuclear localization of OsNLP2 requires the full-length *OsNLP2* coding sequence rather than a specific N-terminal domain, RWP-RK domain, or PB1 domain. These subcellular localization patterns collectively suggest that OsNLP2 is localized in the nucleus, where it may act as a TF and bind to a specific DNA sequence.

### 3.4. ΔOsNLP2 Mutation Triggers Hypersensitive Resistance Reactions against Different M. oryzae Strains Infection

To investigate the roles of *OsNLP2* in plant disease and immunity, *ΔOsnlp2* knockout mutant lines were created from the wild-type rice cultivar DJ by performing T-DNA insertion mutagenesis. T-DNA pGA2715 was inserted at −385 bp in the 5′-untranslated region (UTR) of *OsNLP2* genomic DNA (Figure S3). Two pairs of primers (*OsNLP2* forward and *OsNLP2* reverse; *OsNLP2* forward and T-DNA pGA2715 RB) were designed to select homozygous transgenic *ΔOsnlp2* plants by performing PCR assays with *OsNLP2* genomic DNA (Figure S3, Table S1). The three *ΔOsnlp2* mutant lines (#4-2, #5-1, and #6-4) were confirmed as homozygous mutants by electrophoretic analysis of the PCR products (Figure S3B). The *OsNLP2* transcription levels in wild-type rice DJ and *ΔOsnlp2* mutant lines were analyzed by performing RT-PCR and real-time qRT-PCR assays with an RT primer set (LP and RP). *OsNLP2* expression was significantly suppressed in the three *ΔOsnlp2* mutant lines (Figure S3C). However, all these *ΔOsnlp2* mutant lines grew well normally, as wild-type rice DJ did, which could be used for further experiments. The *ΔOsnlp2* mutant seeds germinated well in the 1/2 MS media to grow to the whole plants (Figure S3D).

To verify that *OsNLP2* is the causal gene for *ΔOsnlp2,* complementation plants were created by transferring the full-length *OsNLP2* coding sequence into *ΔOsnlp2* mutant line #6-4 using the *OsNLP2*:pB2GW7 binary vector (Figure S3A). Levels of *OsNLP2* expression were normalized by invariant expression of the internal control gene *OsUbiquitin*. *OsNLP2* was not expressed in the *ΔOsnlp2* mutant line #6-4. However, real-time qRT-PCR data showed significantly high levels of *OsNLP2* expression in the complementation lines #3, #11, and #12 (Figure S3C). This indicates that *OsNLP2* expression was restored in *ΔOsnlp2* mutant plants.

**Figure 2:**
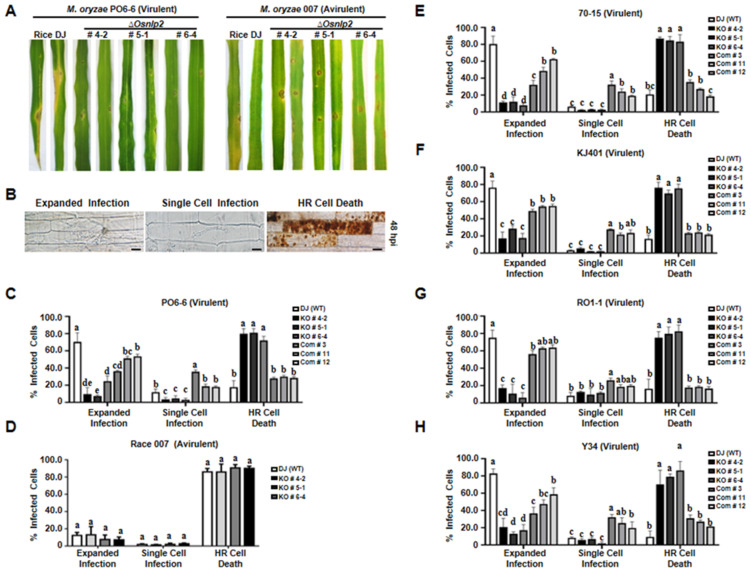
Infection phenotypes of *ΔOsnlp2* mutant lines after inoculation with different *Magnaporthe oryzae* strains. (**A**). Disease symptoms on leaves of rice DJ and *ΔOsnlp2* mutant lines at 5 days after inoculation with *M. oryzae*. *M. oryzae* conidial suspensions (1.0 × 10^5^ conidia mL^−1^) were sprayed on leaves of 3-week-old plants. (**B**). Infection types in epidermal cells of rice leaf sheaths at 48 hpi after *M. oryzae* inoculation. Thin epidermal layers of inoculated rice leaf sheaths were observed under a microscope (Zeiss equipped with Axioplan 2). hpi, hours post inoculation. Scale bars = 10 µm. (**C**–**H**). Quantification of infection types in epidermal cells of rice leaf sheaths at 48 hpi after different *M. oryzae* inoculations. *M. oryzae* strains PO6-6, 007, KJ401, Y34, RO1-1, and 70-15 were used in the pathogenicity test. The cell numbers of different infection types were counted at 48 hpi using a light microscope. The data are means ± standard deviation (SD) of cell numbers of different infection types in leaf sheaths from different rice plants (*n =* 4). Different letters above the bars indicate significantly different means as determined by the least significant difference (LSD) test (*p* < 0.05).

Leaves of 3-week-old wild-type rice DJ and *ΔOsnlp2* knockout mutant lines were spray-inoculated with a conidial suspension (1.0 × 10^5^ conidia mL^−1^) of *M. oryzae* PO6-6 (virulent) and 007 (avirulent). Whole-leaf disease phenotypes were observed and photographed at 5 days after inoculation with *M. oryzae* (Figure 2A). Virulent *M. oryzae* PO6-6 infection caused a typical susceptible disease reaction with expanded large, elliptical, and grayish lesions on rice DJ leaves. By contrast, *ΔOsnlp2* mutant leaves displayed a typical HR-resistant reaction with few small-sized, necrotic, and dark brown spots during *M. oryzae* PO6-6 infection. Avirulent *M. oryzae* 007 infection also induced resistant reactions with some small-sized, brownish lesions in rice DJ and *Osnlp2* mutant leaves (Figure 2A). Mutant *ΔOsnlp2* leaves became discolored, chlorotic, or pale-yellow during *M. oryzae* 007 infection. These infection phenotypes indicate that *ΔOsnlp2* mutants (#4-2, #5-1, and #6-4) are highly resistant to *M. oryzae* PO6-6 and 007.

To examine the early infection responses of *ΔOsnlp2* mutant lines at the cellular level, conidial suspensions (5.0 × 10^5^ conidia mL^−1^) of *M. oryzae* PO6-6 and 007 were inoculated on 5-week-old leaf sheaths of rice DJ and *ΔOsnlp2* mutants. The epidermal layers of infected rice sheaths were excised and observed under a microscope at 48 hpi. The infection responses of rice epidermal cells to *M. oryzae* were classified into the three infection types: (1) expanded infection that develops bulbous invasive hyphae (IH) in a group of cells, (2) single-cell infection with limited thin and filamentous hyphal growth, and (3) HR cell death with cellular aggregates (dark brown color) including vesicles (Figure 2B). Virulent *M. oryzae* PO6-6 caused infection in 70.4% cells but caused HR cell death in 17.6% cells in wild-type rice DJ. By contrast, *M. oryzae* PO6-6 infection caused HR cell death responses in more than 72% of cells in leaf sheaths of the three *ΔOsnlp2* mutant lines (Figure 2C). The complementation lines #3, #11, and #2 exhibited susceptibility to *M. oryzae* strain PO6-6, with 40–60% cells showing expanded infection hyphal growth in multiple cells, and around 20% cells inducing HR cell death. Avirulent *M. oryzae* 007 infection induced HR cell death responses in more than 86% of epidermal cells in both rice DJ and *ΔOsnlp2* mutant lines (Figure 2D). These early infection response data indicate that *ΔOsnlp2* mutants are highly resistant to *M. oryzae* PO6-6 and 007. OsNLP2 is involved in susceptible disease development during virulent *M. oryzae* PO6-6 infection.

We further investigated *ΔOsnlp2* mutant lines’ resistance to different *M. oryzae* strains. KJ401, Y34, RO1-1, and 70-15 are virulent to wild-type rice DJ. The early infection responses in rice leaf sheaths of *ΔOsnlp2* mutants were examined at 48 hpi with different *M. oryzae* strains (Figure 2E–H). Wild-type rice DJ was highly susceptible to all tested *M. oryzae* strains (i.e., 60–80% cells with expanded infection hyphal growth in multiple cells); however, *ΔOsnlp2* mutant lines #4-2, #5-1, and 6-4 were highly resistant to these *M. oryzae* strains, with approximately 80% of the cells showing HR cell death responses at 48 hpi. The complementation lines #3, #11, and #12 were moderately susceptible to all tested *M. oryzae* strains, with 40–60% cells showing expanded infection hyphal growth in epidemic cells. These combined results indicate that *ΔOsnlp2* mutant lines exhibit a broad-spectrum, race-nonspecific resistance to different *M. oryzae* strains.

We investigated whether *OsNLP2* triggered plant cell death by transiently expressing full-length *OsNLP2* and its functional PB1 and RWP-RK-PB1 domains in *N. benthamiana* leaves using agroinfiltration (Figure S4). Transient expression of the positive control Infestin 1 (INF1) distinctly induced a typical cell death response. By contrast, transient expression of full-length *OsNLP2* and PB1 and RWP-RK-PB1 domains did not trigger cell death responses in *N. benthamiana* leaves. The results of this transient expression study indicate that *OsNLP2* expression does not trigger cell death in plants.

### 3.5. ROS-and Iron-Dependent Ferroptotic Cell Death in ΔOsnlp2 Mutants in Response to M. oryzae

**Figure 3:**
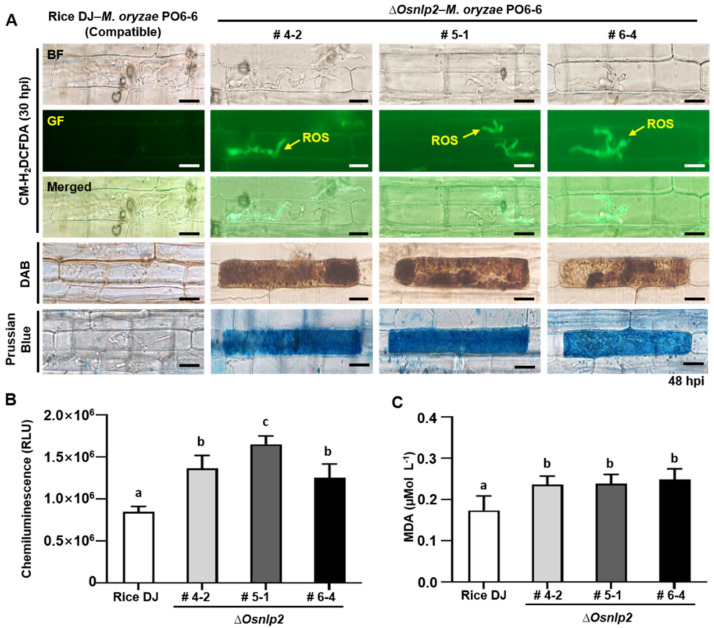
ROS and ferric ion accumulation and lipid peroxidation in leaf sheath cells of rice DJ and *ΔOsnlp2* mutants during virulent *Magnaporthe oryzae* PO6-6 infection. *ΔOsnlp2* KO mutant lines # 4-2, # 5-1, and # 6-4 were used in these experiments. (**A**) Images of ROS and ferric ion accumulation in rice leaf sheath cells. ROS accumulation was detected by CM-H_2_DCFDA and DAB staining, and ferric ion (Fe^3+^) accumulation was visualized by Prussian blue staining. All images were taken using a fluorescence microscope (Zeiss equipped with Axioplan 2) with bright field or GF filters (Ex/Em: 488 nm/505–550 nm wavelengths). BF, bright field; GF, green fluorescence; hpi, hours post inoculation. bars = 10 μm. (**B**) ROS quantification in rice leaf sheaths at 48 hpi. ROS quantities in rice cells were detected by a chemiluminescence assay using a GloMax 96 Microplate Luminometer (Promega, Madison, WI). Values are means ± SD of total relative luminescence units (RLU) (*n* = 10) from different rice sheath discs. (**C**) Determination of lipid peroxidation levels in rice leaf sheaths at 48 hpi. Lipid peroxidation was determined by quantifying malondialdehyde (MDA). Values are means ± SD (*n* = 4) of MDA concentrations in leaf sheath from different plants. Different letters above the bars indicate significantly different means, as determined by the least significant difference (LSD) test (*p* < 0.05).

ROS- and iron-dependent ferroptotic cell death is involved in the defense responses in rice cells infected with avirulent *M. oryzae*. To investigate the cell death triggered by *OsNLP2* knockout mutation, we analyzed ROS and ferric ion (Fe^3+^) accumulation and lipid peroxidation in leaf sheath cells of rice DJ and *ΔOsnlp2* mutants during *M. oryzae* PO6-6 (virulent) and race 007 (avirulent) infection. ROS accumulation was detected and visualized by CM-H_2_DCFDA and DAB staining. The ROS-sensitive CM-H_2_DCFDA dye was used to monitor ROS localization in living plant cells. DAB is oxidized by H_2_O_2_ in the presence of peroxidase to generate a dark brown precipitate in plant cells [34,41]. CM-H_2_DCFDA (GF) and DAB (dark brown) staining showed that ROS (H_2_O_2_) strongly accumulated inside and around IH in wild-type rice DJ cells infected with *M. oryzae* race 007 (avirulent), but not in cells infected with *M. oryzae* PO6-6 (virulent). However, ROS’ strong accumulation could be detected in *ΔOsnlp2* epidermal cells infected with both *M. oryzae* PO6-6 and race 007 (Figure 3A and  Figure S5). CM-H_2_DCFDA-specific ROS-localized fluorescence was distinctly visible inside and around IH in *ΔOsnlp2* mutant cells at 20–48 hpi (Figure S6). DAB-stained epidermal cells with cellular aggregates (dark brown color) were observed at 48 hpi in the leaf sheaths of *ΔOsnlp2* mutants (Figure 3A and  Figure S5). The chemiluminescence assay showed that avirulent *M. oryzae* 007 infection distinctly induced ROS accumulation in both rice DJ and *ΔOsnlp2* mutants to similar levels (Figure S5B). ROS accumulation levels were significantly higher in *ΔOsnlp2* mutant cells than those in rice DJ cells infected with different virulent *M. oryzae* strains at 48 hpi (Figure 3B and  Figure S7).

Ferric ion (Fe^3+^) accumulation and localization in rice epidermal cells were detected by Prussian blue (blue color) staining. Fe^3+^ accumulation at 48 hpi was observed (blue color) inside and around IH in DJ cells in the incompatible interaction with *M. oryzae* race 007 (avirulent), but not in the compatible interaction with *M. oryzae* PO6-6 (virulent). However, Fe^3+^ accumulation could be detected in *ΔOsnlp2* epidermal cells infected with both *M. oryzae* race 007 and PO6-6. (Figure 3A and  Figure S5). These results indicate that ROS and ferric ion (Fe^3+^) simultaneously accumulate in *ΔOsnlp2* cells during *M. oryzae* infection. Low-magnification images of *M. oryzae*-infected-rice leaf sheath cells showed all the infection phenotypes that were not specific to a particular single cell, but commonly detected in the individual constituent cells (Figure S8).

The oxidation of lipids or lipid peroxidation is generated by ROS (hydroxyl radical and hydrogen peroxide) effects on polyunsaturated fatty acids in the membrane, resulting in significant tissue damage [42]. MDA, an indicator of lipid peroxidation, was quantified spectrophotometrically after reaction with TBA [4,43]. We analyzed lipid peroxidation in rice DJ and *ΔOsnpl2* leaf sheaths infected with virulent and avirulent *M. oryzae* strains. Lipid peroxidation (MDA) levels were both induced in DJ and *ΔOsnlp2* cells infected with *M. oryzae* race 007 (Figure S5C). However, it was significantly higher in *ΔOsnlp2* than in DJ cells infected with *M. oryzae* PO6-6 (Figure 3C), indicating that higher ROS and Fe^3+^ levels enhance oxidative stress levels in *ΔOsnlp2* cells. ROS and ferric ion accumulation and the induced cell death indicated the ferroptosis was involved in the defense responses in *ΔOsnlp2* mutant cells during the *M. oryzae* infection process.

### 3.6. Iron Chelator DFO and Ferroptosis Inhibitor Fer-1 Suppress ROS-and Iron-Dependent Ferroptotic Cell Death in ΔOsnlp2 Mutants in Response to M. oryzae

**Figure 4:**
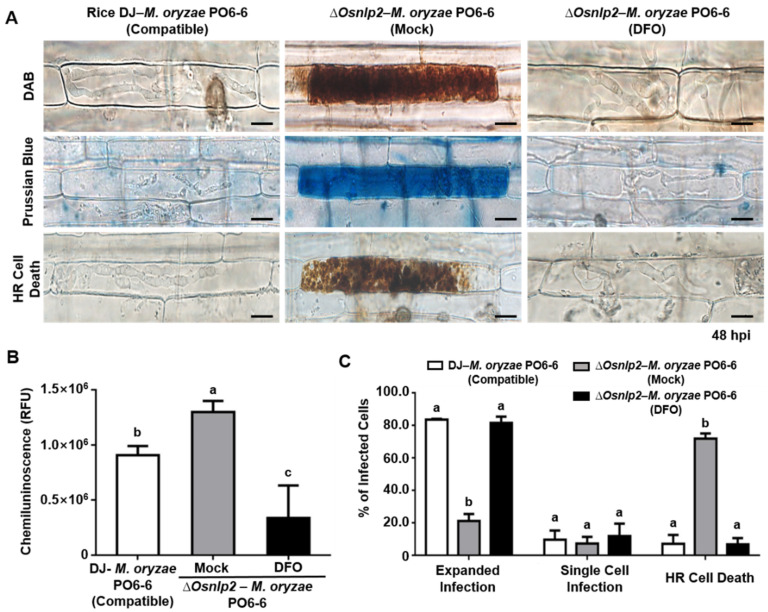
The iron chelator deferoxamine (DFO) suppresses the accumulation of ROS and ferric ions and HR cell death in *ΔOsnlp2* leaf sheaths infected with virulent *Magnaporthe oryzae* PO6-6. (**A**) Inhibition of ROS and ferric ion accumulation and HR cell death in infected rice cells treated with 3 mM DFO. Leaf sheaths of rice DJ and *ΔOsnlp2* mutant line #4-2 were inoculated with *M. oryzae* PO6-6. ROS accumulation was detected by DAB staining, and ferric ion (Fe^3+^) accumulation was visualized by Prussian blue staining. The images shown were taken using a fluorescence microscope (Zeiss equipped with Axioplan 2). hpi, hours post inoculation. Scale bars = 10 μm. (**B**) ROS quantification in infected rice leaf sheaths treated with 3 mM DFO. ROS quantities in rice cells were determined by a chemiluminescence assay. Values are means ± SD of relative luminescence units (RLU) (*n* = 10) from different rice sheath discs. (**C**) Quantification of infection types in rice sheaths treated with 3 mM DFO. The cell numbers of different infection types were counted at 48 hpi using a microscope. The percentages of infected cells are presented as means ± SD from the cell numbers of infection types in rice sheaths of different plants (*n* = 4). Different letters above the bars indicate significantly different means as determined by the least significant difference (LSD) test (*p* < 0.05).

DFO, Fer-1, Cyt A, and DPI affected ferroptotic cell death in wild-type rice Dongjin and Hwayoung in response to *M. oryzae* infection but had no effect on non-infected rice [4]. Hence, these chemicals were used to investigate iron- and ROS-dependent ferroptotic cell death in leaf sheaths of *ΔOsnlp2* mutant and complementation rice plants during *M. oryzae* infection in this study. DFO (3 mM) was applied to leaf sheaths of *ΔOsnlp2* mutant #4-2 after inoculation with virulent *M. oryzae* PO6-6 and avirulent *M. oryzae* 007. ROS accumulation was detected by CM-H_2_DCFDA and DAB staining, and ferric ion accumulation was visualized by Prussian blue staining (Figure 4A and  Figure S9A). ROS and ferric ion accumulation and HR cell death were distinctly suppressed in infected *ΔOsnlp2* leaf sheath cells treated with DFO. The chemiluminescence assay of ROS production showed that DFO treatment significantly suppressed ROS accumulation levels in *ΔOsnlp2* leaf sheaths infected with both *M. oryzae* PO6-6 and race 007 (Figure 4B and  Figure S9B). We quantified the infected cell phenotypes (expanded infection, single-cell infection, and HR cell death) in rice leaf sheaths treated with DFO (3 mM) at 48 hpi (Figure 4C and  Figure S9C). DFO treatment strongly inhibited the HR cell death response in *ΔOsnlp2* leaf sheaths, which ultimately led to the compatible IH growth of *M. oryzae*.

**Figure 5:**
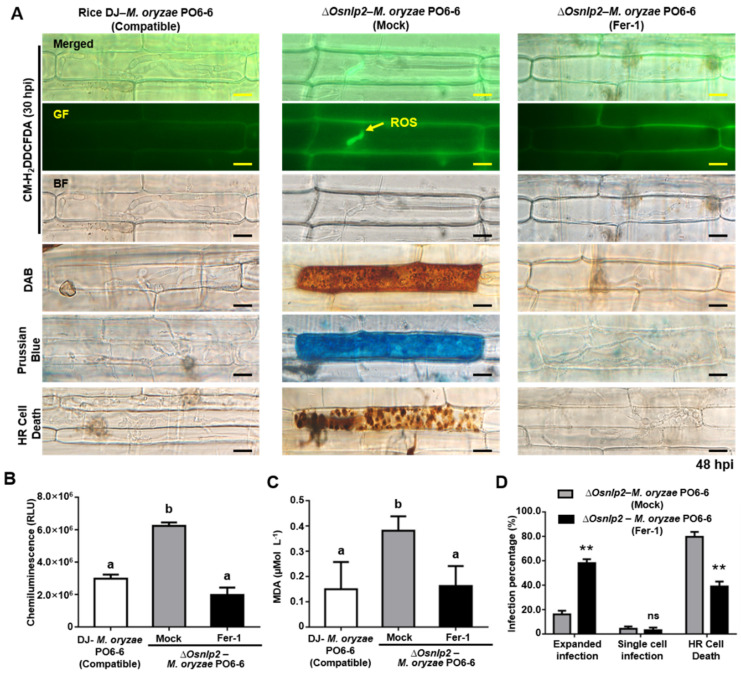
The small-molecule ferroptosis inhibitor ferrostatin-1 (Fer-1) suppresses the accumulation of ROS and ferric ions, lipid peroxidation, and HR cell death in *ΔOsnlp2* leaf sheaths infected with virulent *Magnaporthe oryzae* PO6-6. (**A**) Inhibition of ROS and ferric ion accumulation and HR cell death in infected rice cells treated with 10 μM ferrostatin-1. Leaf sheaths of rice DJ and *ΔOsnlp2* mutant line #4-2 were inoculated with *M. oryzae* PO6-6. ROS accumulation was detected by CM-H_2_DCFDA and DAB staining, and ferric ion (Fe^3+^) accumulation was visualized by Prussian blue staining. The images shown were taken using a fluorescence microscope (Zeiss equipped with Axioplan 2) with bright field or GF filters (Ex/Em: 488 nm/505–550 nm wavelengths). BF, bright field; GF, green fluorescence; hpi, hours post inoculation. Scale bars = 10 μm. (**B**) ROS quantification in infected rice leaf sheaths treated with 10 μM ferrostatin-1. ROS quantities in rice cells were determined by a chemiluminescence assay. Values are means ± SD of relative luminescence units (RLU) (*n* = 10) from different rice sheath discs. (**C**) Determination of lipid peroxidation levels in infected rice sheaths treated with 10 μM ferrostatin-1. Lipid peroxidation was determined by quantifying malondialdehyde (MDA). The values are presented as means ± SD (*n* = 4) of MDA concentrations in leaf sheaths from different plants. (**D**) Quantification of infection types in rice leaf sheaths treated with 10 μM ferrostatin-1. The cell numbers of different infection types were counted at 48 hpi using a light microscope. The percentages of infected cells are presented as means ± SD from the cell numbers of infection types in rice sheaths of different plants (*n* = 4). Different letters above the bars indicate significantly different means as determined by the least significant difference (LSD) test (*p* < 0.05). Asterisks above the bars indicate significant differences as determined by Student’s *t*-test (*p* < 0.01).

The *ΔOsnlp2* mutant line #4-2 was treated with the ferroptosis inhibitor ferrostatin-1 (Fer-1) (10 μM). At 24 hpi, Fer-1 distinctly inhibited the accumulation of ROS and ferric ions in *ΔOsnlp2* leaf sheath epidermal cells inside and around IH during *M. oryzae* PO6-6 (virulent) or 007 (avirulent) infection (Figure 5A and  Figure S9A). Fer-1 treatment significantly inhibited HR cell death responses around IH with cellular aggregates (dark brown color) in *ΔOsnlp2* leaf sheaths, which led to successful colonization of IH (Figure 5A,D). ROS production was significantly suppressed in *ΔOsnlp2* leaf sheath epidermal cells after Fer-1 treatment (Figure 5B and  Figure S9B). Lipid peroxidation levels in mock- and Fer-1–treated *ΔOsnlp2* leaf sheath cells were tested at 48 hpi using MDA quantification (Figure 5C). Avirulent and Virulent *M. oryzae* infection distinctly induced lipid peroxidation in *ΔOsnlp2* leaf sheath cells, which displayed an HR cell death response. However, treatment with 10 μM Fer-1 suppressed lipid peroxidation in *ΔOsnlp2* leaf sheaths infected with *M. oryzae* strains.

### 3.7. The Actin Polymerization Inhibitor Cyt A and NADPH Oxidase Inhibitor Diphenyleneiodonium (DPI) Suppress ROS and Ferric Ion Accumulation and HR Cell Death in ΔOsnlp2 Leaf Sheaths in Response to M. oryzae

**Figure 6:**
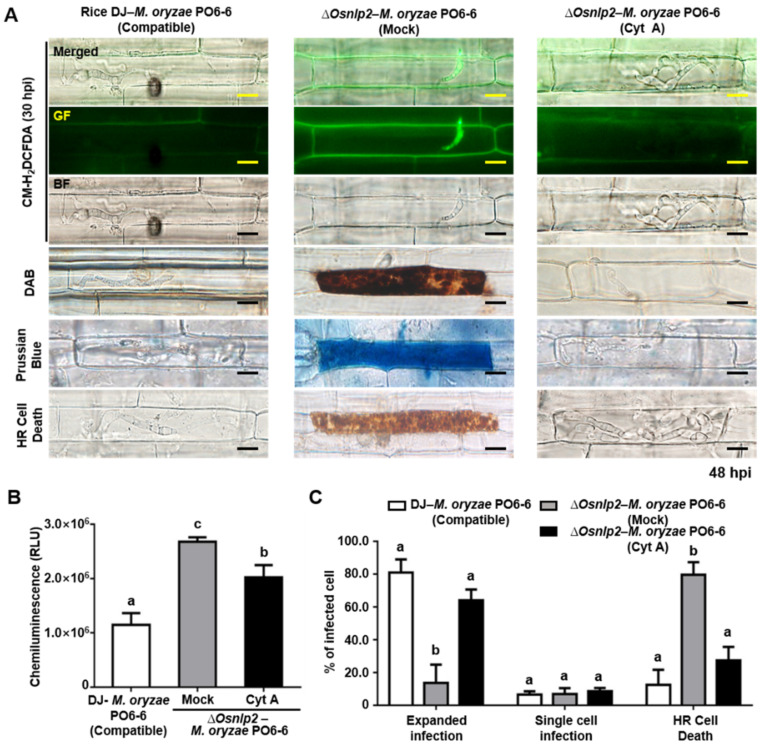
The actin filament inhibitor cytochalasin A (Cyt A) suppresses the accumulation of ROS and ferric ions and HR cell death in *ΔOsnlp2* leaf sheaths infected with virulent *Magnaporthe oryzae* PO6-6. (**A**) Inhibition of ROS and ferric ion accumulation and HR cell death in infected rice cells treated with 20 μg mL^−1^ Cyt A. Leaf sheaths of rice DJ and *ΔOsnlp2* mutant line #4-2 were inoculated with *M. oryzae* PO6-6. ROS accumulation was detected by CM-H_2_DCFDA and DAB staining, and ferric ion (Fe^3+^) accumulation was visualized by Prussian blue staining. The images were taken using a fluorescence microscope (Zeiss equipped with Axioplan 2) with bright field or GF filters (Ex/Em: 488 nm/505–550 nm wavelengths). BF, bright field; GF, green fluorescence; hpi, hours post inoculation. Scale bars = 10 μm. (**B**) ROS quantification in infected rice leaf sheaths treated with 20 μg mL^−1^ cytochalasin A. ROS quantities in rice cells were determined by a chemiluminescence assay. Values are means ± SD of relative luminescence units (RLU) (*n* = 10) from different rice sheath discs. (**C**) Quantification of infection types in rice leaf sheaths treated with 20 μg mL^−1^ cytochalasin A. The cell numbers of different infection types were counted at 48 hpi using a light microscope. The percentages of infected cells are presented as means ± SD from the cell numbers of infection types in leaf sheaths of different rice plants (*n* = 4). Different letters above the bars indicate significantly different means as determined by the least significant difference (LSD) test (*p* < 0.05).

**Figure 7:**
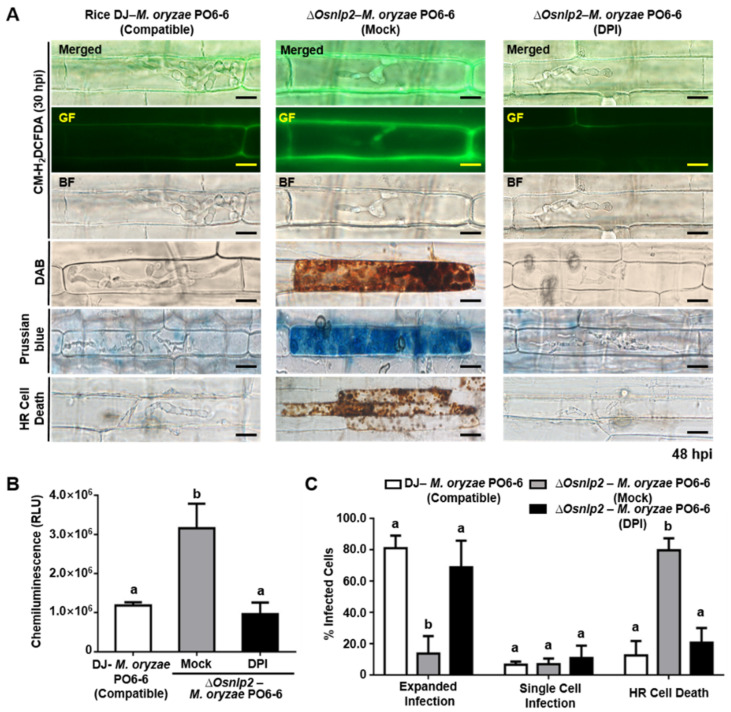
The NADPH oxidase inhibitor diphenyleneiodonium (DPI) suppresses the accumulation of ROS and ferric ions and HR cell death in *ΔOsnlp2* leaf sheaths infected with virulent *Magnaporthe oryzae* PO6-6. (**A**) Inhibition of ROS and ferric ion accumulation and HR cell death in infected rice cells treated with 5 μM DPI. Leaf sheaths of rice DJ and *ΔOsnlp2* mutant line #4-2 were inoculated with *M. oryzae* PO6-6. ROS accumulation was detected by CM-H_2_DCFDA and DAB staining, and ferric ion (Fe^3+^) accumulation was visualized by Prussian blue staining. The images shown were taken using a fluorescence microscope (Zeiss equipped with Axioplan 2) with bright field or GF filters (Ex/Em: 488 nm/505–550 nm wavelength). BF, bright field; GF, green fluorescence; hpi, hours post inoculation. Scale bars = 10 μm. (**B**) ROS quantification in infected rice leaf sheaths treated with 5 μM DPI. ROS quantities in rice cells were determined by a chemiluminescence assay. Values are means ± SD of relative luminescence units (RLU) (*n* = 10) from different rice sheath discs. (**C**) Quantification of infection types in rice leaf sheaths treated with 5 μM DPI. The cell numbers of different infection types were counted at 48 hpi using a light microscope. The percentages of infected cells are presented as means ± SD from the cell numbers of infection types in leaf sheaths of different rice plants (*n* = 4). Different letters above the bars indicate significantly different means as determined by the least significant difference (LSD) test (*p* < 0.05).

Conidial suspension (5 × 10^5^ conidia mL^−1^) of virulent *M. oryzae* PO6-6 or avirulent *M. oryzae* 007 with Cyt A (20 µg mL^−1^) or DPI (5 µM) solution was inoculated on rice leaf sheaths. Cyt A treatment suppressed the *M. oryzae*-induced accumulation of ROS and Fe^3+^ and HR cell death in *ΔOsnlp2* leaf sheath cells, as shown in the images of CM-H_2_DCFDA, DAB, and Prussian blue staining (Figure 6A and  Figure S9A). The chemiluminescence assay showed that ROS production in *ΔOsnlp2* leaf sheaths was significantly suppressed at 48 hpi after Cyt A treatment (Figure 6B and  Figure S9B). Both virulent and avirulent *M. oryzae* strains grew well with normal hyphal structures in *ΔOsnlp2* leaf sheath cells treated with Cyt A (20 µg mL^−1^) (Figure 6A,C). DPI (5 µM) distinctly inhibited the accumulation of ROS and ferric ions and HR cell death in *ΔOsnlp2* leaf sheaths infected with *M. oryzae* PO6-6 or *M. oryzae* 007, leading to successful IH colonization (Figure 7 and  Figure S9). ROS and iron accumulation were hardly to be detected in *ΔOsnlp2* cells after DPI treatment (Figure 7A and  Figure S9A). Chemiluminescence quantification indicated that DPI significantly suppressed ROS production in *ΔOsnlp2* leaf sheaths at 48 hpi (Figure 7B and  Figure S9B). DPI treatment distinctly promoted *M. oryzae* PO6-6 and *M. oryzae* 007 infection but inhibited HR cell death in *ΔOsnlp2* leaf sheaths (Figure 7C and  Figure S9C).

### 3.8. Time-Course Expression of Defense-Related Genes in ΔOsnlp2 Mutant Cells during M. oryzae Infection

**Figure 8:**
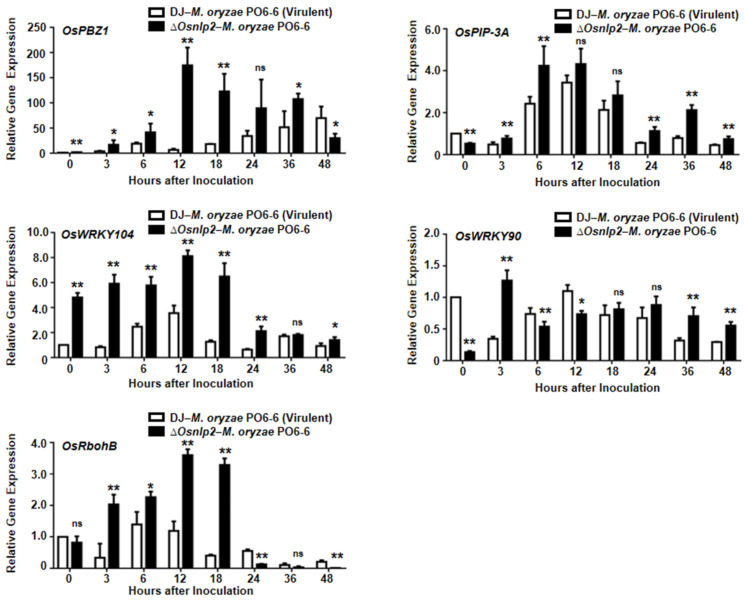
Real-time qRT-PCR analysis of time-course expression of some defense-related genes in leaf sheaths of rice DJ and *ΔOsnlp2* mutant (#4-2) plants during virulent *Magnaporthe oryzae* PO6-6 infection. Relative expression levels of *OsPBZ1*, *OsPIP-3A*, *OsWRKY104*, *OsWRKY90*, and *OsRbohB* at different time points after inoculation were obtained by normalizing with respect to the expression levels of the internal control *OsUbiquitin*. The data are means ± SD of relative gene expression levels in rice leaf sheaths from three independent experiments. Asterisks indicate significant differences as determined by Student’s *t*-test (*p* < 0.05, *p* < 0.01). ns, not significant.

To analyze the resistant responses, several defense-related genes expression levels in leaf sheaths of DJ and *ΔOsnlp2* mutant line #4-2 were detected at different time points after inoculation with virulent *M. oryzae* PO6-6 and race 007 (Figure 8 and  Figure S10). *OsPBZ1* was identified as a PBZ-inducible gene in rice [44]. In the incompatible interaction between DJ and *M. oryzae* race 007, *OsPBZ1* was distinctly induced at 3–36 hpi in DJ cells, but not in the compatible interaction between DJ and *M. oryzae* PO6-6. However, *OsPBZ1* was also induced at 3–36 hpi in *ΔOsnlp2* mutant cells (Figure 8 and  Figure S10). Plasma membrane intrinsic proteins (PIPs), aquaporins, are membrane channels that facilitate the transport of water and small neutral molecules across biological membranes in living organisms, having a crucial role in the regulation of water transport and plant growth [45,46]. *OsPIP-3A* was highly induced at 6 and 24–48 hpi in *ΔOsnlp2* mutant line #4-2 compared with DJ inoculated with *M. oryzae* PO6-6 (Figure 8). WRKY proteins are a large family of TFs involved in various plant processes, especially in inducible defense responses to biotic and abiotic stresses [47]. *OsWRKY104* induction was apparent in leaf sheaths of *ΔOsnlp2* mutant line #4-2 at 3–24 hpi. By contrast, *OsWRKY90* was only induced in DJ inoculated with avirulent *M. oryzae* race 007, but not in *ΔOsnlp2* leaf sheaths inoculated with *M. oryzae* PO6-6, which was different with *OsWRKY104* (Figure 8 and  Figure S10). Plant RBOHs produce ROS that are involved in plant immunity [3,48]. The NADPH oxidase *OsRbohB* was distinctly induced at 3–18 hpi in *ΔOsnlp2* leaf sheaths (Figure 8). These combined results indicate that *OsNLP2* disruption triggers *OsPBZ1*, *OsPIP-3A*, *OsWRKY104*, and *OsRbohB* expression in *ΔOsnlp2* leaf sheaths during the *M. oryzae* infection process.

## 4. Discussion

### 4.1. Subcellular Localization and Functional Analysis of OsNLP2 Domains

Plant-specific RWP-RK family proteins are TFs that bind to specific DNA sequences (cis-elements) in the promoter regions adjacent to the genes that they regulate [49,50]. In this study, we screened a truncated RWP-RK domain-containing protein from the rice cDNA library. The NIN-like protein OsNLP2 was identified using DNA sequence BLAST in NCBI. OsNLP2 contains an N-terminal GAF domain, RWP-RK domain, and C-terminal PB1 domain. GAF domains are ubiquitous motifs present in cGMP-specific phosphodiesterases, adenylyl cyclases, and bacterial transcription factor FhlA [51]. The DNA-binding function of the RWP-RK domain determines its nuclear localization in plant cells. As expected, the subcellular localization study of OsNLP2:GFP showed that OsNLP2 was localized to the nucleus of *N. benthamiana* cells (Figure 1). The full-length OsNLP2, but not a specific N-terminal domain, RWP-RK domain, or PB1 domain, moved to the cellular nuclei. These results suggest that OsNLP acts as a TF that binds to a specific DNA sequence to regulate gene expression in rice. RWP-RK proteins have been proposed to be, at least partly, localized in the nucleus [21]. The RWP-RK motif (Arg-Trp-Pro-X-Arg-Lys, where X indicates any amino acid) binds to the nitrate-responsive *cis*-element (NRE) of nitrate-inducible genes [23,52,53]. The PB1 domain is responsible for protein–protein interactions [23,40]. The PB1 domain:GFP of OsNLP2 was localized to the cytoplasm and plasma membrane, which may mediate protein–protein interactions associated with nitrate-inducible gene expression in rice. Recently, Konishi and Yanagisawa [23] demonstrated that NLP–NLP interactions mediated by the PBI domain occur in a variety of different *Arabidopsis* NLP TFs.

### 4.2. OsNLP2 Expression Positively Regulates Susceptible Disease Development

A plentiful supply of nitrogen (N) is required for fungal pathogen growth, but increased nitrogen supply causes disease susceptibility in plants [18,54,55]. Rice NLP proteins, such as *OsNLP1* and *OsNLP4*, enhance NUE and yield in rice [22,27]. *OsNLP1*, a key gene regulating nitrogen (N) utilization, was rapidly induced by nitrogen starvation in rice [27]. In rice, *OsNLP3* is induced after germination and repressed by heat treatment [21]. *OsNLP4* is repressed by several abiotic stresses and induced by low phosphate availability [21]. However, the question whether *OsNLP* genes function in plant disease and immunity has not been investigated. In our present study, virulent *M. oryzae* PO6-6 infection distinctly induced *OsNLP2* expression and caused susceptible disease symptoms in rice DJ. By contrast, avirulent *M. oryzae* 007 infection did not trigger *OsNLP2* induction in rice DJ, which subsequently led to HR-resistance reactions with necrotic and brownish spots. These infection phenotypes suggest that *OsNLP2* induction is involved in susceptible blast disease and suppresses HR cell death and defense responses in rice during *M. oryzae* infection. *OsNLP2* induction seems likely to create a beneficial nutrition condition for *M. oryzae* growth. Namely, OsNLP2 may act as a positive regulator of blast disease development during *M. oryzae* infection.

### 4.3. ΔOsnlp2 Mutants Exhibit ROS-and Iron-Dependent Ferroptotic HR Cell Death Responses to M. oryzae Infection

We recently reported iron- and ROS-dependent ferroptosis in incompatible rice–*M. oryzae* interactions [4,5]. In the present study, we used *ΔOsnlp2* mutant lines to investigate whether *OsNLP2* was involved in ROS- and iron-dependent ferroptotic HR cell death responses to *M. oryzae* infection. *ΔOsnlp2* mutants showed typical HR cell death and resistant phenotypes to *M. oryzae* PO6-6, which is virulent to wild-type rice DJ. ROS bursts during pathogen infection are required for HR cell death and immunity and disease-related cell death [3,56,57]. ROS and ferric ion accumulation and lipid peroxidation occurred in *ΔOsnpl2* leaf sheaths. Ferric ion accumulation in *M. oryzae*-infected rice cells may stimulate ROS production, such as hydrogen peroxide (H_2_O_2_) and hydroxyl radicals (·OH), which degrade DNA and other biomolecules [13]. Distinct focal ROS accumulation was detected around *M. oryzae* IH in *ΔOsnlp2* mutant cells, which ultimately led to ROS- and iron-dependent ferroptotic cell death by Fe^3+^ accumulation and lipid peroxidation. These results suggest that *OsNLP2* mutation triggers ferroptotic cell death and HR-resistant reactions against *M. oryzae* infection. An iron-dependent ROS burst and lipid peroxidation may mediate ferroptotic cell death in rice [4,5]. Liu et al. [36] previously demonstrated that ferric iron deposition at the powdery mildew infection site mediates the ROS burst in epidermal cells of wheat (*Triticum aestivum*) leaves.

### 4.4. DFO, Fer-1, Cyt A, and DPI Inhibit ROS-and Iron-Dependent Ferroptotic Cell Death in ΔOsnlp2 Mutants

The iron chelator deferoxamine (DFO) prevents iron-dependent ferroptotic cell death in mammalian cells [9,58]. During *M. oryzae* PO6-6 or 007 infection, DFO treatment inhibited the accumulation of ROS and ferric ions and HR cell death, in *ΔOsnlp2* leaf sheaths, which may become susceptible to *M. oryzae* invasion. At that time, *M. oryzae* could not take up DFO-chelated iron from the host *ΔOsnlp2* leaf sheath cells, as suggested by Dangol et al. (2019) [4]. The accumulation of lipid-based ROS such as lipid peroxides is involved in the iron-dependent ferroptotic cell death [14]. In this study, the lipid-based ROS scavenger ferrostatin-1 (Fer-1) distinctly inhibited lipid peroxidation and ferroptotic HR cell death in *ΔOsnlp2* leaf sheaths. Fer-1 may inhibit the production of lipid hydroperoxides by blocking lipid peroxidation [9,59]. These combined results suggest that *Osnlp2* mutation triggers iron- and ROS-dependent lipid peroxidation to induce ferroptotic HR cell death.

Cytochalasin (Cyt) binds to actin microfilaments and inhibits actin polymerization in plant cells [60,61,62]. Cyt A treatment distinctly suppressed the accumulation of ROS and Fe^3+^ and HR cell death, leading to normal *M. oryzae* growth in *ΔOsnlp2* leaf sheath cells. Cyt A may interfere with iron accumulation in the *M. oryzae* infection site in *ΔOsnlp2* leaf sheath cells. Actin microfilament polymerization is required for the deployment of key early plant defense responses [62,63,64]. Diphenyleneiodonium (DPI) inhibits cellular ROS production mediated by NADPH oxidases in the plasma membrane [65,66,67]. NADPH oxidases, known as respiratory burst oxidase homologs (RBOHs), are responsible for ROS production in plants during pathogen infection [3,68,69]. DPI treatment distinctly suppressed ROS and ferric ion accumulation and HR cell death in *ΔOsnpl2* mutant cells [4]. A possible inhibition of NADPH oxidase OsRbohB by DPI may suppress the iron-dependent accumulation of ROS and HR cell death in rice during *M. oryzae* infection. All DFO, Fer-1, Cyt A, and DPI treatments attenuated ferroptotic cell death and promoted *M. oryzae* infection in *ΔOsnlp2* mutants. Collectively, these data suggest that *OsNLP2* expression negatively regulates iron-dependent ROS accumulation, lipid peroxidation, and ferroptotic HR cell death during *M. oryzae* infection.

### 4.5. Broad-Spectrum, Nonspecific Resistance of ΔOsnlp2 Mutants to Different M. oryzae Strains

In this study, *OsNLP2* mutation induced *OsPBZ1*, *OsPIP-3A*, *OsWRKY104*, and *OsRbohB* in *ΔOsnlp2* leaf sheaths during *M. oryzae* infection. Probenazole (PBZ) induces race-nonspecific resistance in rice plants against *M. oryzae*. *OsPBZ1* is known as a PBZ-inducible gene in rice [44]. Plasma membrane intrinsic proteins (PIPs), aquaporins, function as membrane channels to mediate H_2_O_2_ transport inside the plant cell across the plasma membrane [3,45]. OsPIP2;1 has a crucial role in the regulation of water transport and plant growth [46]. The rice genome contains more than 100 WRKY genes. The OsWRKY71 TF is involved in rice defense response [70]. The NADPH oxidase OsRbohB exhibits apoplastic ROS-producing activity in rice cells [17]. The distinct induction of defense-related genes, such as *OsPBZ1*, *OsPIP-3A*, *OsWRKY104*, and *OsRbohB*, by the *OsNLP2* mutation may trigger iron- and ROS-dependent ferroptotic cell death and broad-spectrum resistance in *ΔOsnlp2* mutants during *M. oryzae* infection.

*ΔOsnlp2* mutant lines were highly resistant to different *M. oryzae* strains KJ401, Y34, RO1-1, and 70-15, which are virulent to wild-type rice DJ. A complementation test verified that *OsNLP2* is the causal gene for *ΔOsnlp2* mutant phenotypes. The complementation lines exhibited susceptible infection types, i.e., 40–60% cells with expanded infection hyphal growth of different *M. oryzae* strains. These data support the hypothesis that loss-of-function of *OsNLP2* may trigger broad-spectrum, non-specific resistance to diverse *M. oryzae* strains. To investigate whether the OsNLP2 mutation affects plant growth, we measured parameters of *ΔOsnlp2* and wild-type rice DJ plants. There was no significant difference in the number of spikes, the number of kernels per spikes, and the weight of one thousand seeds (data not shown). These results demonstrated that the OsNLP2 mutation induced enhanced resistance without compromising growth and yield. In this study, we have screened OsNLP2 from rice cDNA library by yeast two-hybrid screening using a *M. oryzae* effector as bait. A partial, but not entire, OsNLP2 protein was found to interact with the *M. oryzae* effector Avr-Pikm (data not shown). This suggests the possibility that *M. oryzae* effectors interact with OsNLP proteins inside rice cells. Furthermore, different *M. oryzae* strains may secrete non-specific effectors to induce *OsNLP2* expression, which ultimately leads to create a beneficial cellular condition for *M. oryzae* infection and suppress HR cell death and defense responses in rice.

*OsNLP2* mutation triggered the expression of some defense-related genes, such as *OsPBZ1*, *OsPIP-3A*, *OsWRKY104*, and *OsRbohB*, in *ΔOsnlp2* mutants. Rice defense-related genes, which function in pattern-triggered immunity (PTI) signaling, have been proposed to mediate broad-spectrum resistance to two or more pathogen species independently [71]. A natural allele of the C_2_H_2_-type TF BSR-D1 in rice confers broad-spectrum resistance to *M. oryzae* [72]. Zhou et al. [73] recently demonstrated that the rice *bsr-k1* (broad-spectrum resistance Kitaake-1) mutant confers broad-spectrum resistance against *M. oryzae* and *Xanthomonas oryzae* pv. *oryzae*. Loss-of- function of the *Bsr-k1* gene induced *OsPAL1* expression in the *bsr-k1* mutant. *OsPAL1* overexpression in wild-type rice conferred resistance to *M. oryzae*. Collectively, our study suggests that OsNLP2 negatively regulates ferroptotic cell death and immune responses in rice, and provides a valuable gene source for molecular breeding of rice with broad-spectrum resistance to diverse *M. oryzae* strains.

## 5. Conclusions

In this study, we identified that *OsNLP2* was highly induced during the virulent *M. oryzae* infection process but suppressed during avirulent *M. oryzae* infection. OsNLP2 was localized in the nucleus, suggesting that OsNLP2 function as a transcriptional factor regulating the rice defense response. The *ΔOsnlp2* lines exhibited an immune response with hypersensitive cell death against the virulent *M. oryzae* strains. However, the complementation lines of *OsNLP2* restored the susceptible phenotype to the virulent *M. oryzae* strains. Deletion of *OsNLP2* induced non-race specific resistance to virulent *M. oryzae* strains. ROS and ferric ions are highly accumulated in *ΔOsnlp2* cells to ensure the strong resistance to virulent *M. oryzae* strains. Small molecules of DFO, Fer-1, Cyt A, and DPI suppressed the ferroptotic cell death in *ΔOsnlp2* cells during *M. oryzae* infection. The defense-related genes *OsPBZ1*, *OsPIP-3A*, *OsRbohB* and *OsWRKY104* were highly induced to ensure broad-spectrum resistance in *ΔOsnlp2* cells. In summary, OsNLP2 acts as a negative regulator of ferroptotic cell death and defense responses in rice with broad-spectrum resistance to rice blast disease.

## Data Availability

All of the data is contained within the article and the Supplementary Materials.

## Supplementary Materials

The following supporting information can be downloaded at: https://www.mdpi.com/article/10.3390/antiox11091795/s1. Figure S1: Amino acid sequence alignment of GAF-like domain, RWP-RK domain, and PB1 domain of rice NLP family proteins with other plant NLP proteins using MEGA7 software. Figure S2: Phylogenetic tree of rice NLP family proteins with other plant NLP proteins. Figure S3: *ΔOsnlp2* mutant and complementary lines genotyping. Figure S4: *Agrobacterium*-mediated transient expression of OsNLP2 and its PB1 and RWP-RK-PB1 domains does not trigger cell death in *Nicotiana benthamiana* cells. Figure S5: ROS and ferric ion accumulation and lipid peroxidation in leaf sheath cells of rice DJ and *ΔOsnlp2* mutants during avirulent *Magnaporthe oryzae* 007 infection. Figure S6: ROS accumulation in rice DJ and *ΔOsnlp2* mutant cells at different time points after inoculation with *Magnaporthe oryzae* PO6-6. Figure S7: ROS quantification in leaf sheaths of rice DJ and *ΔOsnlp2* mutant lines infected with different *Magnaporthe oryzae* strains. Figure S8: Low-magnification images of ROS and ferric ion accumulation and cell death response in leaf sheath cells of rice DJ and *ΔOsnlp2* mutant during virulent *Magnaporthe oryzae* PO6-6 infection. Figure S9: Suppression of HR cell death in *ΔOsnlp2* leaf sheaths infected with avirulent *Magnaporthe oryzae* 007 after treatment with deferoxamine (DFO), ferrostatin-1 (Fer-1), cytochalasin A (Cyt A), and diphenyleneiodonium (DPI). Figure S10: Real-time qRT-PCR analysis of time-course expression of defense-related genes in leaf sheaths of rice DJ and *ΔOsnlp2* mutant plants infected with *Magnaporthe oryzae* PO6-6 (virulent) and 007 (avirulent). Table S1: Primers used in this study. Table S2: Accession numbers of RWP-PK proteins used in this study.
