# Supplementary material for: The NIN-Like Protein OsNLP2 Negatively Regulates Ferroptotic Cell Death and Immune Responses to Magnaporthe oryzae in Rice
Source: Antioxidants (Basel). 2022 Sep 12;11(9):1795. doi: 10.3390/antiox11091795 (PMC9495739; doi:10.3390/antiox11091795)
Supplement: Supplementary file 1 [file antioxidants-11-01795-s001.zip › antioxidants-1888378-supplementary/Supplementary Files/supplementary tables.pdf]

Table S1. Primers used in this study.

Gene name		Sequence (5′-3′)	Description
OsNLP2	F-1	AAAAAGCAGGCTTCATGGATATGCCTACG	Subcellular localization
	R-1	AGAAAGCTGGGTTTATGAGCTATGTGC	
RWP-RK domain	F-1	AAAAAGCAGGCTTCATGAATATTAGCTTG	
PB1 domain	F-1	AAAAAGCAGGCTTCATGCTGACAGTTAAG	
OsNLP2	F-2	CGACGACAAGACCCTATGGATATGCCTACGCCA	Transient expression
	R-2	GAGGAGAAGAGCCCTTTATGAGCTATGTGCCGC	
RWP-RK domain	F-2	CGACGACAAGACCCTATGAATATTAGCTTGGAT	
PB1 domain	F-2	CGACGACAAGACCCTATGCTGACAGTTAAGGCA	
OsNLP2 RT	LP	GCATCAAGCCACCCTACCTT	Transcription analysis
	RP	TCACGATTCGCGACCCTATG	
OsNLP2	FP	ATGGATATGCCTACGCCATC	Complementary construction
	RP	CTTGAGGTCCATTCTGGCACCCCA	
OsNLP2	F	GAGCCAAGGAATCTCCTCCTCTTC	Genotyping
	R	CTTGAGGTCCATTCTGGCACCCCA	
T-DNA 2715	RB	GTTACGTCCTGTAGAAACCCCAA	
OsPBZ1	F	GCTACAGGCATCAGTGGTCA	qRT-PCR
	R	GACTCAAACGCCACGAGAAT	
OsPIP 3A	F	TCATCCTCGTCTACACCGTC	
	R	CACCCAGAAGATCCAGTGGT	
OsWRKY90	F	ATGGCCAGCAGTAGCGACCATG	
	R	CTCTCTGTTTCTCTCAAACTGA	
OsWRKY104	F	TGCCATTACTTCCGAGCGAC	
	R	ATTGTGAGGTGCTTGAGCCA	
OsRbohB	F	GAATTCATGGCTGACCTGGAAGCAGGCA	
	R	CTCGAGTTAGAAGTTCTCCTTGTGGAAA	
OsUbiquitin	F	GTGGTGGCCAGTAAGTCCTC	
	R	GGACACAATGATTAGGGATCA	

*F, forward; R, reverse; LP, left primer; RP, right primer; FP, forward primer; RB, right border.

Table S2. Accession numbers of NLP proteins used in this study.

Plant	Gene name	Accession number
Arabidopsis thaliana	AtNLP1	AT2G17150
	AtNLP2	AT4G35270
	AtNLP3	AT4G38340
	AtNLP4	AT1G20640
	AtNLP5	AT1G76350
	AtNLP6	AT1G64530
	AtNLP7	AT4G24020
	AtRKD1	AT1G18790
	AtRKD2	AT1G74480
	AtRKD3	AT5G66990
	AtRKD4	AT5G53040
	AtRKD5	AT4G35590
Medicago truncatula	MtNLP1	Medtr2g099350
	MtNLP2	Medtr4g068000
	MtNLP3	Medtr1g100970
Brachypodium distachyon	BdNLP1	Bradi1g76340
	BdNLP2	Bradi4g37147
	BdNLP3	Bradi 4g20717 & Bradi4g20730
	BdNLP4	Bradi5g23300
	BdNLP5	Bradi3g03170
	BdNLP6	Bradi2g31710
	BdNLP7	Bradi2g08177
Oryza sativa ssp. *japonica*	OsNLP1	Os03g03900
	OsNLP2	Os04g41850
	OsNLP3	Os01g13540
	OsNLP4	Os09g37710
	OsNLP5	Os11g16290
	OsNLP6	Os02g04340
	OsRKD1	Os01g14420
	OsRKD3	Os01g37100
	OsRKD4	Os04g47640
	OsRKD5	Os06g12360
	OsRKD6	Os02g51090
	OsRKD7	Os08g19820
	OsRKD8	Os12g12970
	OsRKD9	Os09g27190
	OsRKD10	Os02g20530
	OsPBZ1	Os12g36880
	OsPIP 3A	Os09g36930
	OsWRKY90	Os09g30400
	OsWRKY104	Os11g02520
	OsRbohB	Os01g25820
	OsUbiquitin	Os06g46770
