# Supplementary material for: The NIN-Like Protein OsNLP2 Negatively Regulates Ferroptotic Cell Death and Immune Responses to Magnaporthe oryzae in Rice
Source: Antioxidants (Basel). 2022 Sep 12;11(9):1795. doi: 10.3390/antiox11091795 (PMC9495739; doi:10.3390/antiox11091795)
Supplement: Supplementary file 1 [file antioxidants-11-01795-s001.zip › antioxidants-1888378-supplementary/Supplementary Files/Supplemetary figures.pdf]

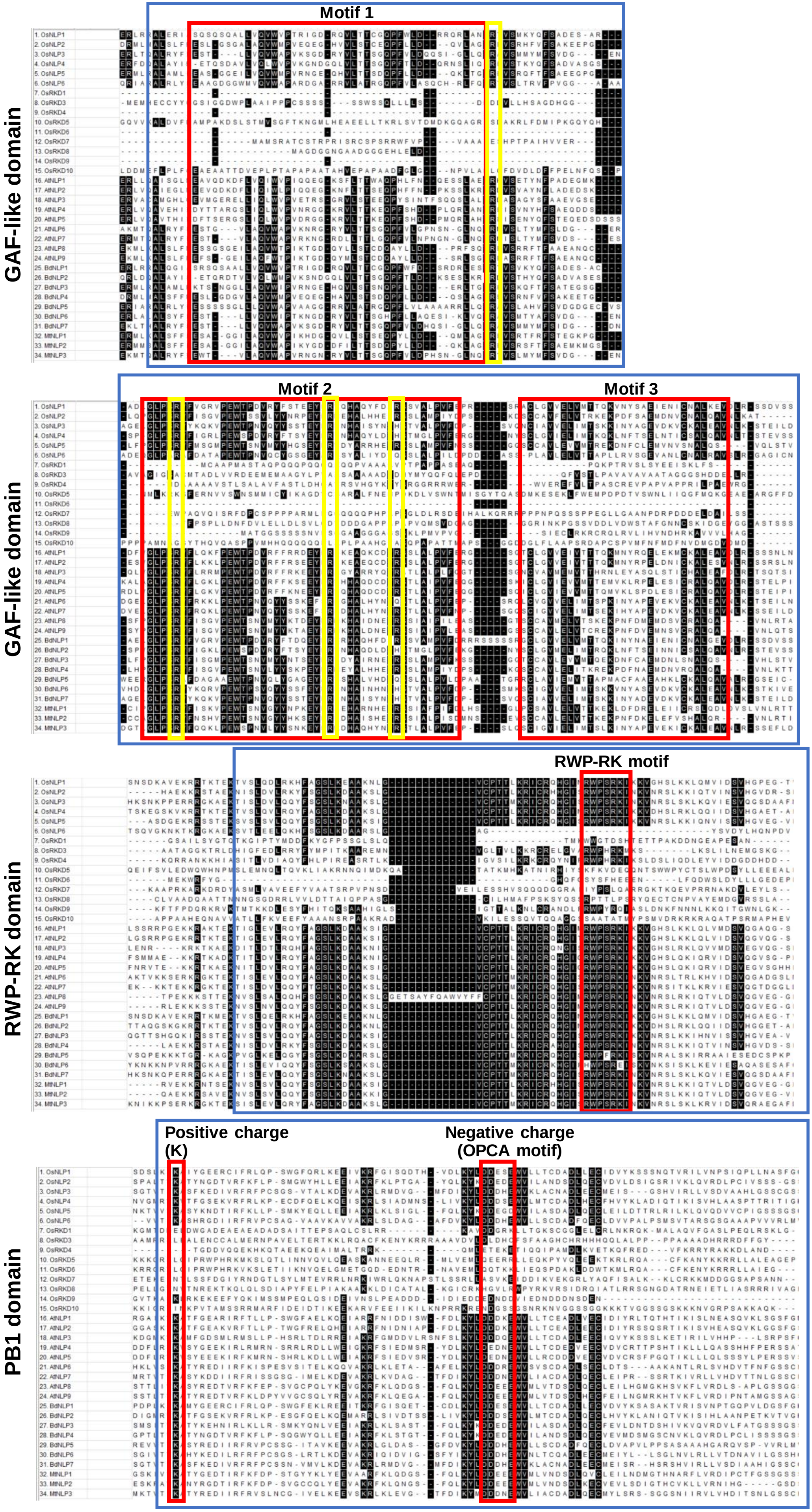

Motif 1
GAF-like domain
Motif 2
Motif 3
GAF-like domain
RWP-RK motif
RWP-RK domain
Positive charge
(K)
Negative charge
(OPCA motif)
PB1 domain

Figure S1. Amino acid sequence alignment of GAF-like domain, RWP-RK domain, and PB1 domain of rice NLP family proteins with other plant NLP proteins using MEGA7 software. Black shading represents identity and similarity in at least 70% of the sequences aligned. (Upper) Red boxes are the GAF-like motifs; blue boxes represent the GAF-like domains in NLP proteins; and yellow boxes represent the conserved Arg, which is essential for cGMP binding. (Middle) Red box is the RWP-RK motif, and blue box is the RWP-RK domain in NLP proteins. (Lower) Red box is the conserved lysine (K) and OPCA motifs; blue boxes represent the PB1 domains in NLP proteins. Sequence used in protein alignment can be found in the Rice Genome Project website (http://rice.plantbiology.msu.edu/), the Arabidopsis Information Resource (TAIR, http://www.arabidopsis.org/), Phytozome (http://www.phytozome.net/), and NCBI (https://www.ncbi.nlm.nih.gov/). Accession numbers of the aligned plant NPL proteins are listed in Supplementary Table S2. NLP, NIN-like protein; RKD, RWP-RK domain-containing proteins; GAF, found in cGMP-specific phosphodiesterases, adenylyl cyclases, and FhlA.

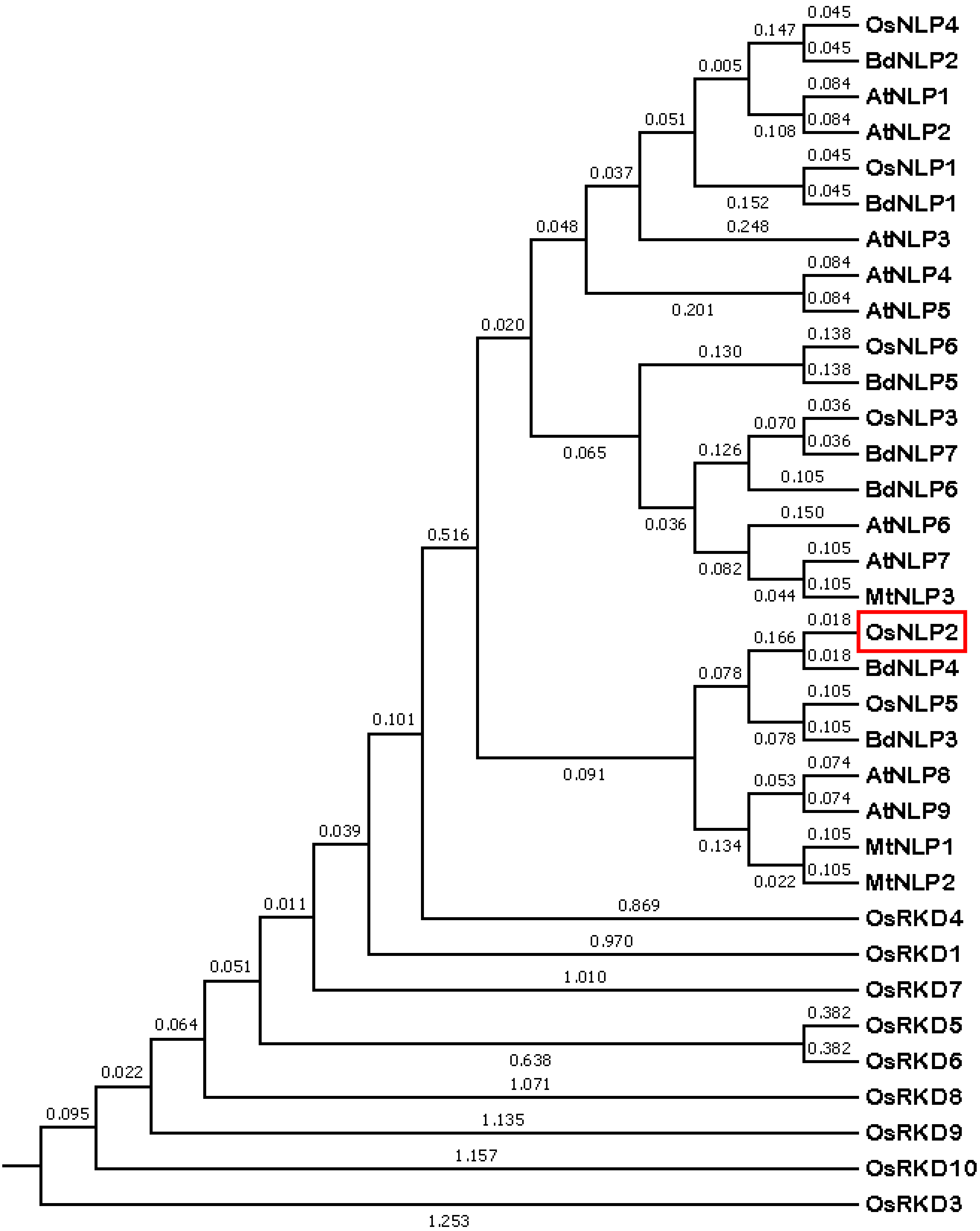
OsNLP4
BdNLP2
AtNLP1
AtNLP2
OsNLP1
BdNLP1
AtNLP3
AtNLP4
AtNLP5
OsNLP6
BdNLP5
OsNLP3
BdNLP7
BdNLP6
AtNLP6
AtNLP7
MtNLP3
OsNLP2
BdNLP4
OsNLP5
BdNLP3
AtNLP8
AtNLP9
MtNLP1
MtNLP2
OsRKD4
OsRKD1
OsRKD7
OsRKD5
OsRKD6
OsRKD8
OsRKD9
OsRKD10
OsRKD3

Figure S2. Phylogenetic tree of rice NLP family proteins with other plant NLP proteins. Phylogenetic tree was constructed using the neighbor-joining method and MEGA7 software. Accession numbers of the aligned plant NPL proteins are listed in Supplementary Table S2. NLP, NIN-like protein; RKD, RWP-RK domain-containing proteins.

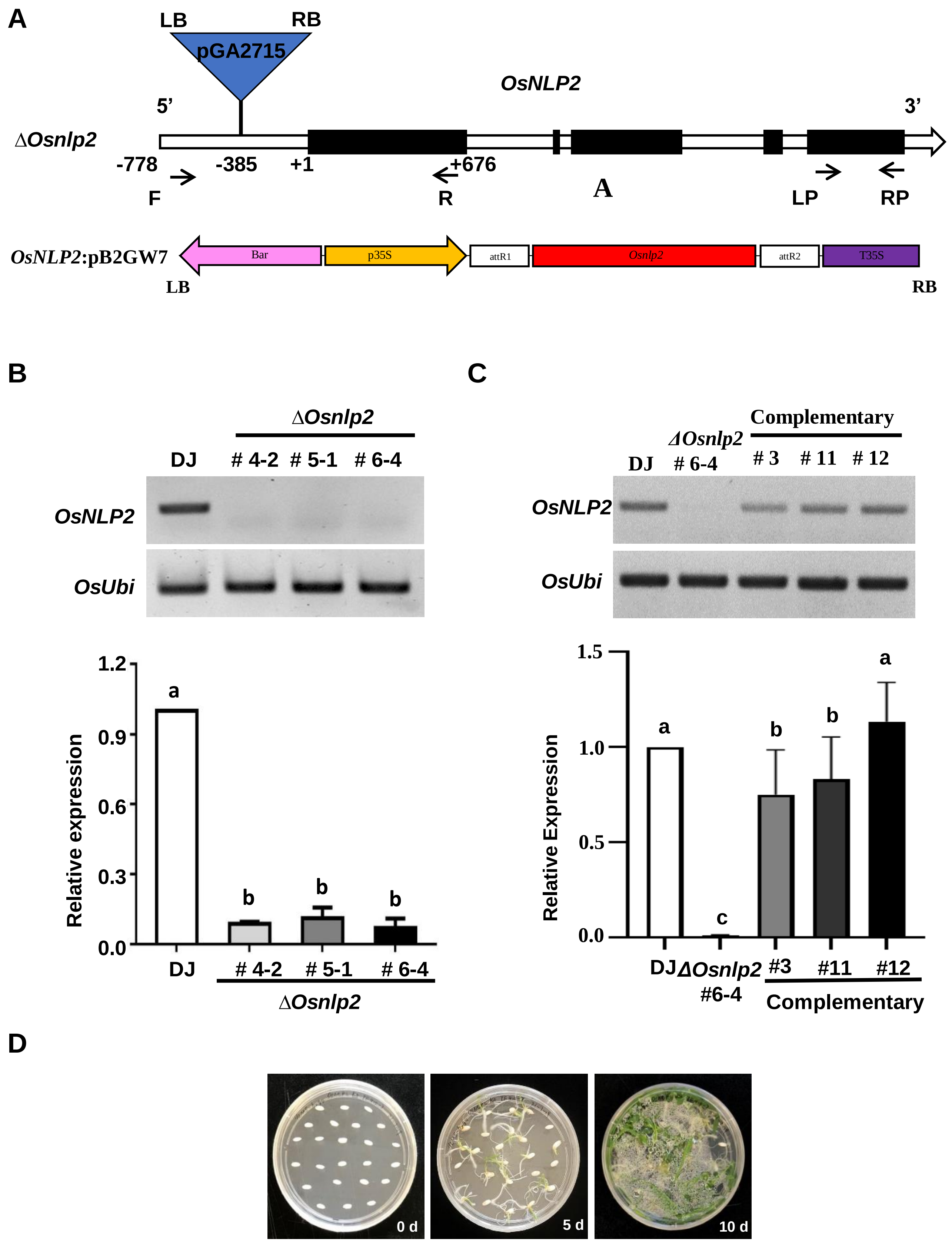

ΔOsnlp2 # 6-4

Seed Germination (%)			
Repeat Ⅰ	Repeat Ⅱ	Repeat Ⅲ	Average
72.92	66.67	79.17	72.92±6.25

Figure S3. *ΔOsnlp2* mutant and complementary lines genotyping. (A) Schematic representation of *ΔOsnlp2* mutants and complementary lines . The *OsNLP2* gene contains five exons and four introns, which were depicted by open boxes and solid boxes, respectively. *ΔOsnlp2* mutants were constructed using the T-DNA insertion method. T-DNA pGA2715 was inserted into the 5′-UTR region (–385 bp site) of *OsNLP2*. The primers F (forward) and R (reverse) were selected at –778 and +676 bp, respectively. T-DNA RB (right border) primer sequence was supplied by the RiceGE (http://signal.salk.edu./cgi-bin/RiceGE). The primer pair RT forward (LP) and RT reverse (RP) were used for gene expression in the RT-PCR assay. Black arrows indicated the primer directions. LP, left primer; RP, right primer; LB, left border; RB, right border. (B) *OsNLP2* expression in rice DJ and *ΔOsnlp2* mutants. Rice *Ubiquitin* (*OsUbi*) transcript levels were used to normalize the transcript level of *OsNLP2*. The data are presented as means ± SD of relative expression quantities of *OsNLP2* in leaf sheaths from different rice plants (n=4). Different letters above the bars indicated significantly different means as determined by the least significant difference (LSD) test (P<0.05). (C) *OsNLP2* expression in leaf sheaths of *ΔOsnlp2* complementation plants. Rice *Ubiquitin* (*OsUbi*) transcript levels were used to normalize the transcript levels of *OsNLP2*. The data are presented as means ± SD of relative expression quantities of *OsNLP2* in leaf sheaths from different rice plants (n=4). Different letters above the bars indicated significantly different means as determined by the least significant difference (LSD) test (P<0.05). (D) Germination of T1 *ΔOsnlp2* seeds. The sterilized seeds were germinated in one-half MS media containing 30 mg L^{-1} hygromycin. The seed germination was monitored after incubation for 10 days in the MS media. The pictures were taken at 0, 5, and 10 days after incubation. The average percentages of seed germination are represented as means ± SD. The experiments were independently repeated three times.

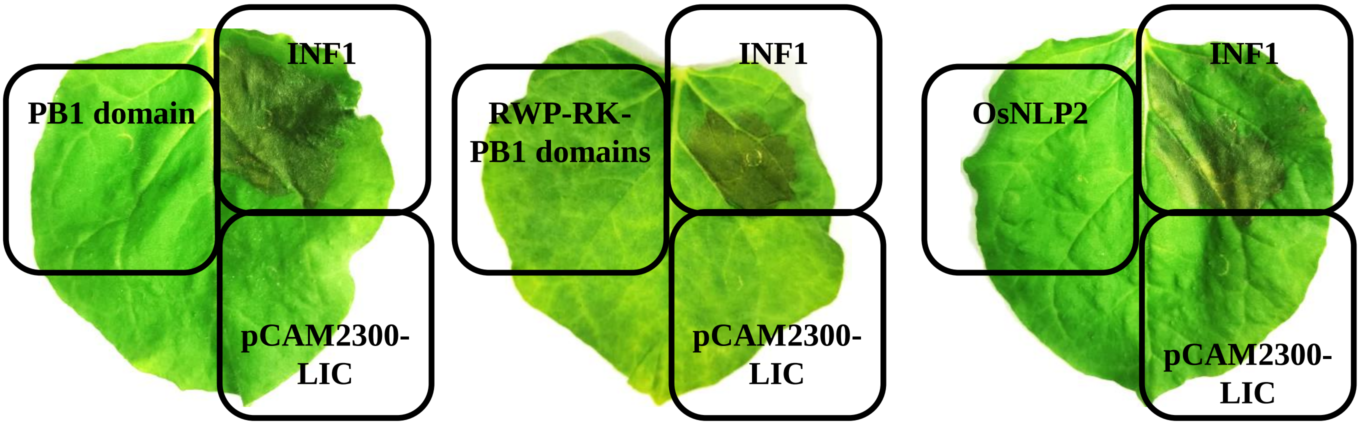
INF1
PB1 domain
pCAM2300-
LIC
INF1
RWP-RK-
PB1 domains
pCAM2300-
LIC
INF1
OsNLP2
pCAM2300-
LIC

Figure S4. *Agrobacterium*-mediated transient expression of *OsNLP2* and its PB1 and RWP-RK-PB1 domains does not trigger cell death in *Nicotiana benthamiana* cells. *Agrobacterium* GV3101 cultures (OD_{600}=1.0) were transformed with *OsNLP2* and its domains, and were infiltrated into *N. benthamiana* leaves. Agrobacterium cultures with INF1:pCAM2300-LIC and empty vector pCAM2300-LIC were used as positive and negative controls, respectively. Cell death images were taken at 48 h after agroinfiltration into *N. benthamiana* leaves.

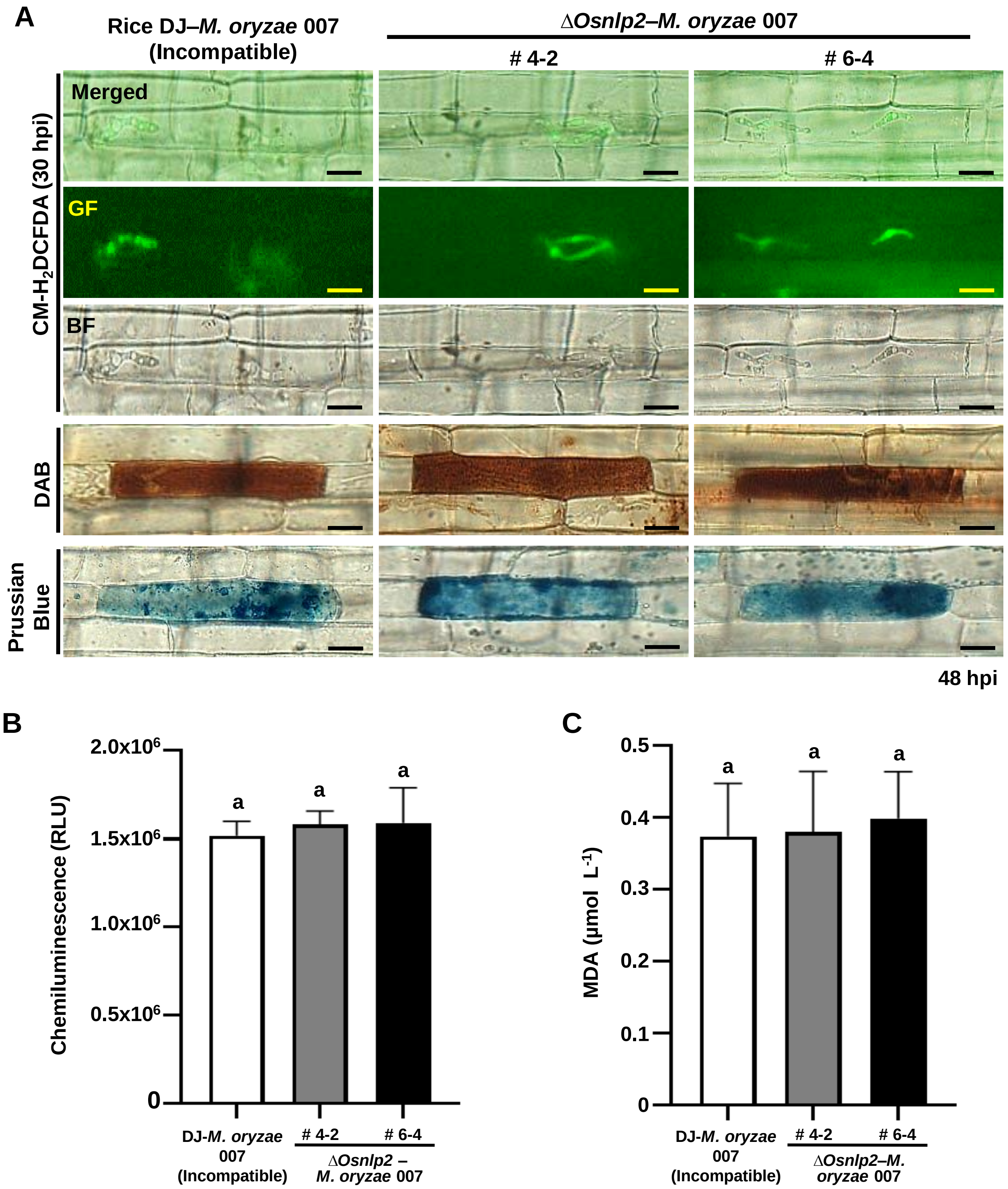
A
Rice DJ–M. oryzae 007
(Incompatible)
ΔOsnlp2–M. oryzae 007
4-2
6-4
CM-H2DCFDA (30 hpi)
Merged
GF
BF
DAB
Prussian Blue
48 hpi
B
Chemiluminescence (RLU)
2.0x10^6
1.5x10^6
1.0x10^6
0.5x10^6
0
a
a
a
DJ-M. oryzae 007 (Incompatible)
4-2
6-4
ΔOsnlp2 – M. oryzae 007
C
MDA (μmol L-1)
0.5
0.4
0.3
0.2
0.1
0
a
a
a
DJ-M. oryzae 007 (Incompatible)
4-2
6-4
ΔOsnlp2–M. oryzae 007

Figure S5. ROS and ferric ion accumulation and lipid peroxidation in leaf sheath cells of rice DJ and *ΔOsnlp2* mutants during avirulent *Magnaporthe oryzae* 007 infection. (A) Images of ROS and ferric ion accumulation in rice leaf sheath cells. ROS accumulation was detected by CM-H_2DCFDA and DAB staining, and ferric ion (Fe^{3+}) accumulation was visualized by Prussian blue staining. All images were taken using a fluorescence microscope (Zeiss equipped with Axioplan 2) with bright field or GF filters (Ex/Em: 488 nm/505–550 nm wavelength). BF, bright field; GF, green fluorescence; hpi, hours post inoculation. Scale bars = 10 μm. (B) ROS quantification in rice leaf sheaths during infection. ROS quantities in rice cells were detected by chemiluminescence assay using a GloMax 96 Microplate Luminometer (Promega, Madison, WI). Values are means ± SD of total relative luminescence units (RLU) (n=10) from different rice sheath discs. (C) Determination of lipid peroxidation levels in rice leaf sheaths during infection. Lipid peroxidation was determined by quantifying malondialdehyde (MDA). Values are presented as means ± SD (n=3) of MDA concentrations in leaf sheaths from different plants. Letters above the bars indicated significantly different means, as determined by the least significant difference (LSD) test (P<0.05).

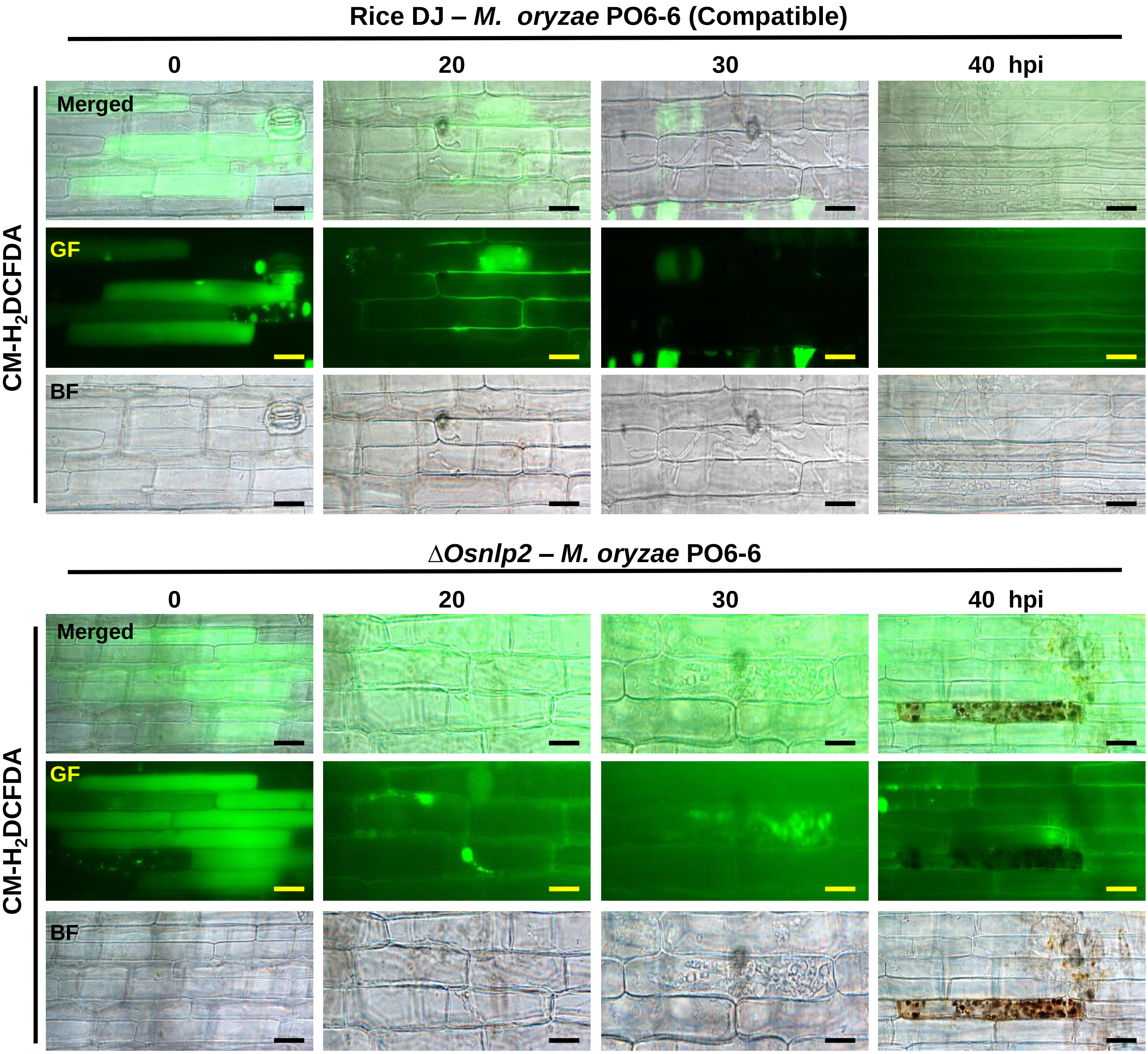

Rice DJ – M. oryzae PO6-6 (Compatible)
0
20
30
40 hpi
Merged
GF
BF
CM-H2DCFDA
ΔOsnlp2 – M. oryzae PO6-6
0
20
30
40 hpi
Merged
GF
BF
CM-H2DCFDA

Figure S6. ROS accumulation in rice DJ and *ΔOsnlp2* mutant cells at different time points after inoculation with *Magnaporthe oryzae* PO6-6. CM-H_2DCFDA (GF) staining reveals ROS accumulation in rice cells. The images were taken using a fluorescence microscope (Zeiss equipped with Axioplan 2) with a bright field or GF filters (Ex/Em: 488 nm/505–550 nm wavelength). BF, bright field; GF, green fluorescence; hpi, hours post inoculation. Scale bars=10 μm.

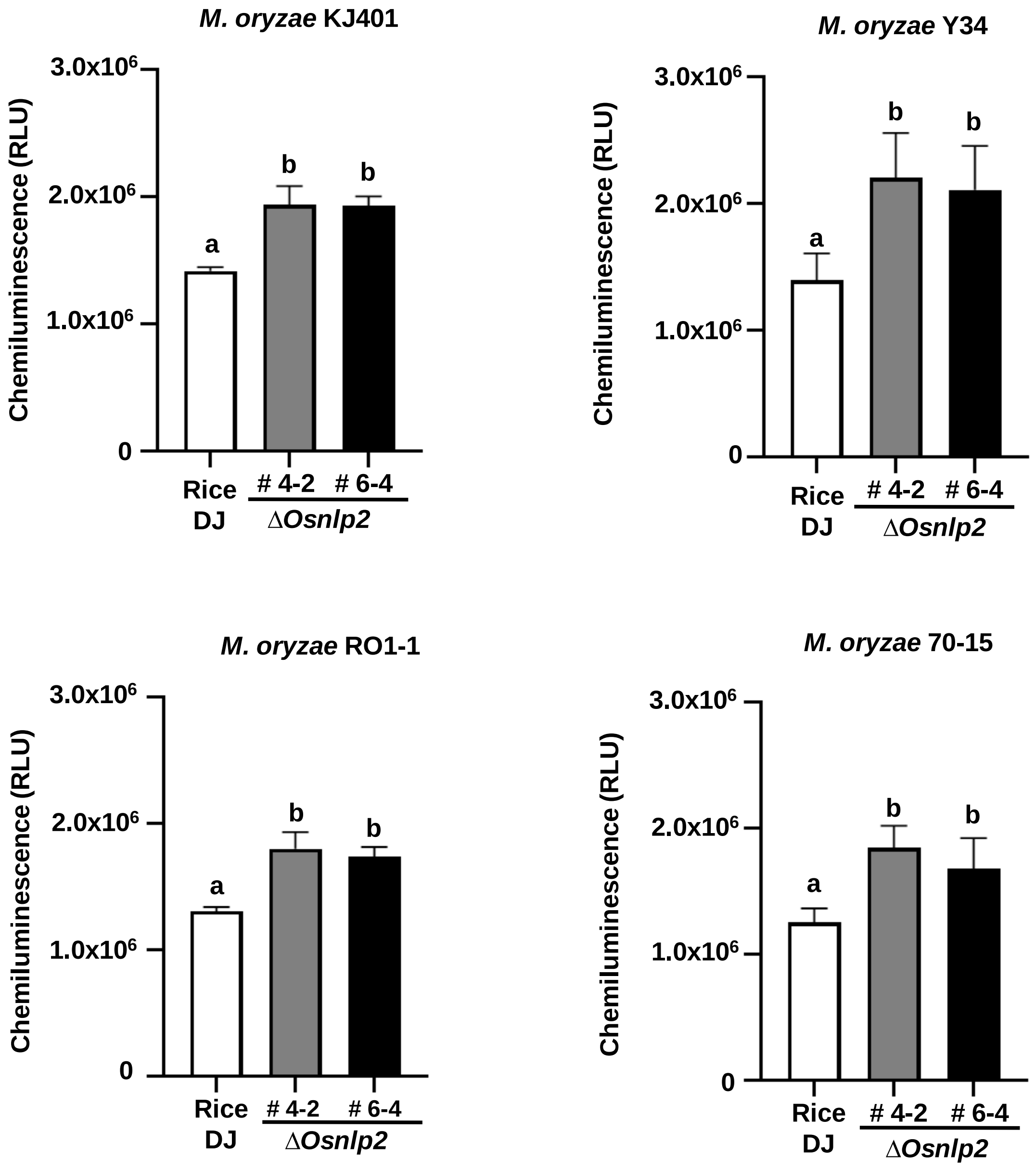
M. oryzae KJ401
M. oryzae Y34
M. oryzae RO1-1
M. oryzae 70-15
Chemiluminescence (RLU)
3.0x10^6
2.0x10^6
1.0x10^6
0
a
b
Rice DJ
4-2
6-4
ΔOsnlp2

Figure S7. ROS quantification in leaf sheaths of rice DJ and *ΔOsnlp2* mutant lines infected with different *Magnaporthe oryzae* strains. Leaf sheaths of rice DJ and *ΔOsnlp2* mutant lines (# 4-2 and # 6-4) were inoculated with *M. oryzae* strains KJ401, Y34, RO1-1, and 70-15. ROS quantities in rice cells were determined by a chemiluminescence assay. Values are means ± SD of relative luminescence units (RLU) (n=10) from different rice sheath discs. Different letters above the bars indicated significantly different means as determined by the least significant difference (LSD) test (P<0.05).

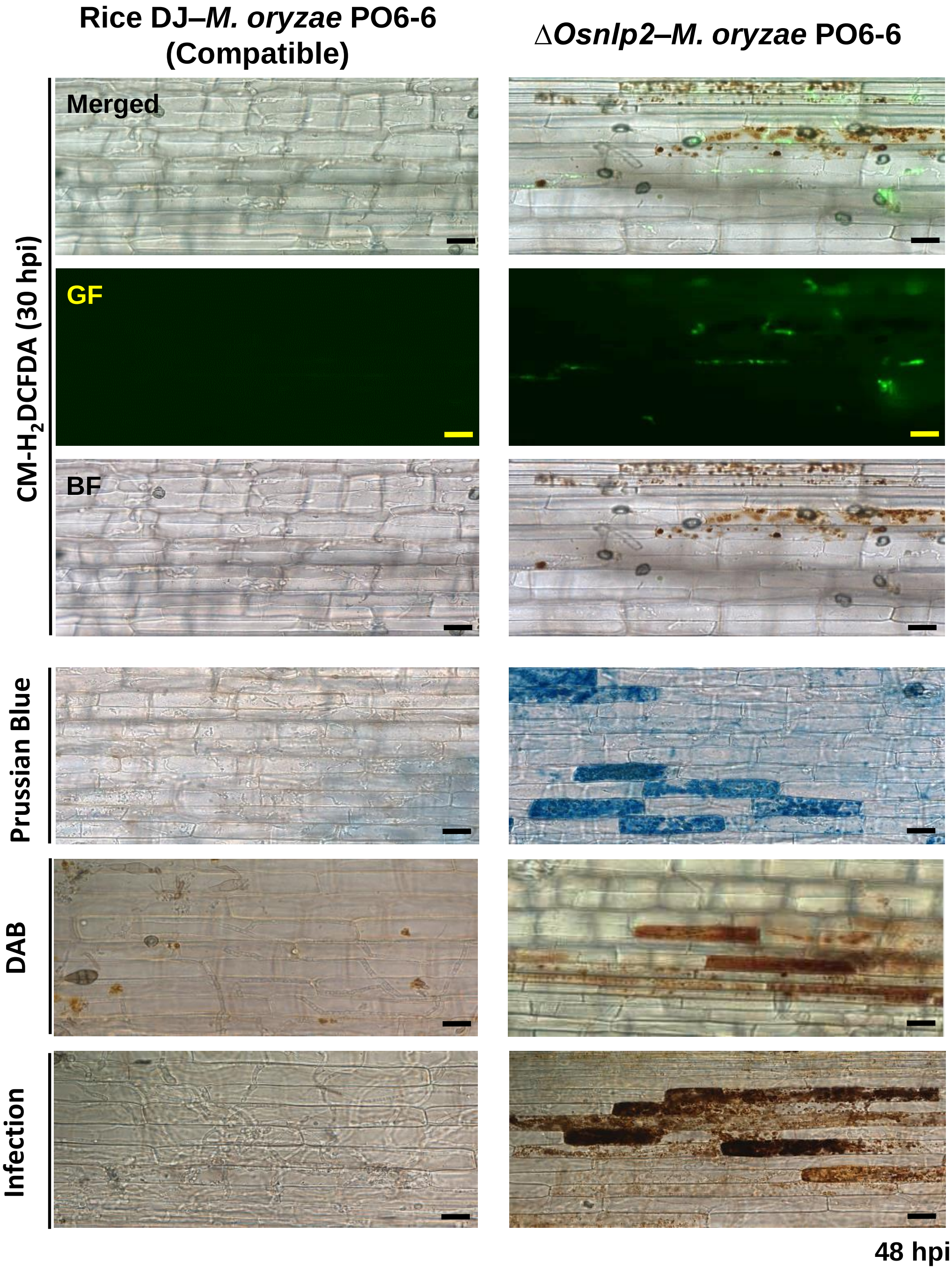

Rice DJ–*M. oryzae* PO6-6 (Compatible)
∆*Osnlp2*–*M. oryzae* PO6-6
CM-H2DCFDA (30 hpi)
Merged
GF
BF
Prussian Blue
DAB
Infection
48 hpi

Figure S8. Low magnification images of ROS and ferric ion accumulation and cell death response in leaf sheath cells of rice DJ and *ΔOsnlp2* mutant during virulent *Magnaporthe oryzae* PO6-6 infection. ROS accumulation was detected by CM-H_2DCFDA and DAB staining, and ferric ion (Fe^{3+}) accumulation was visualized by Prussian blue staining. All images were taken using a fluorescence microscope (Zeiss equipped with Axioplan 2) with BF or GF filters (Ex/Em: 488 nm/505–550 nm wavelength). BF, bright field; GF, green fluorescence; hpi, hours post inoculation. Scale bars=10 μm.

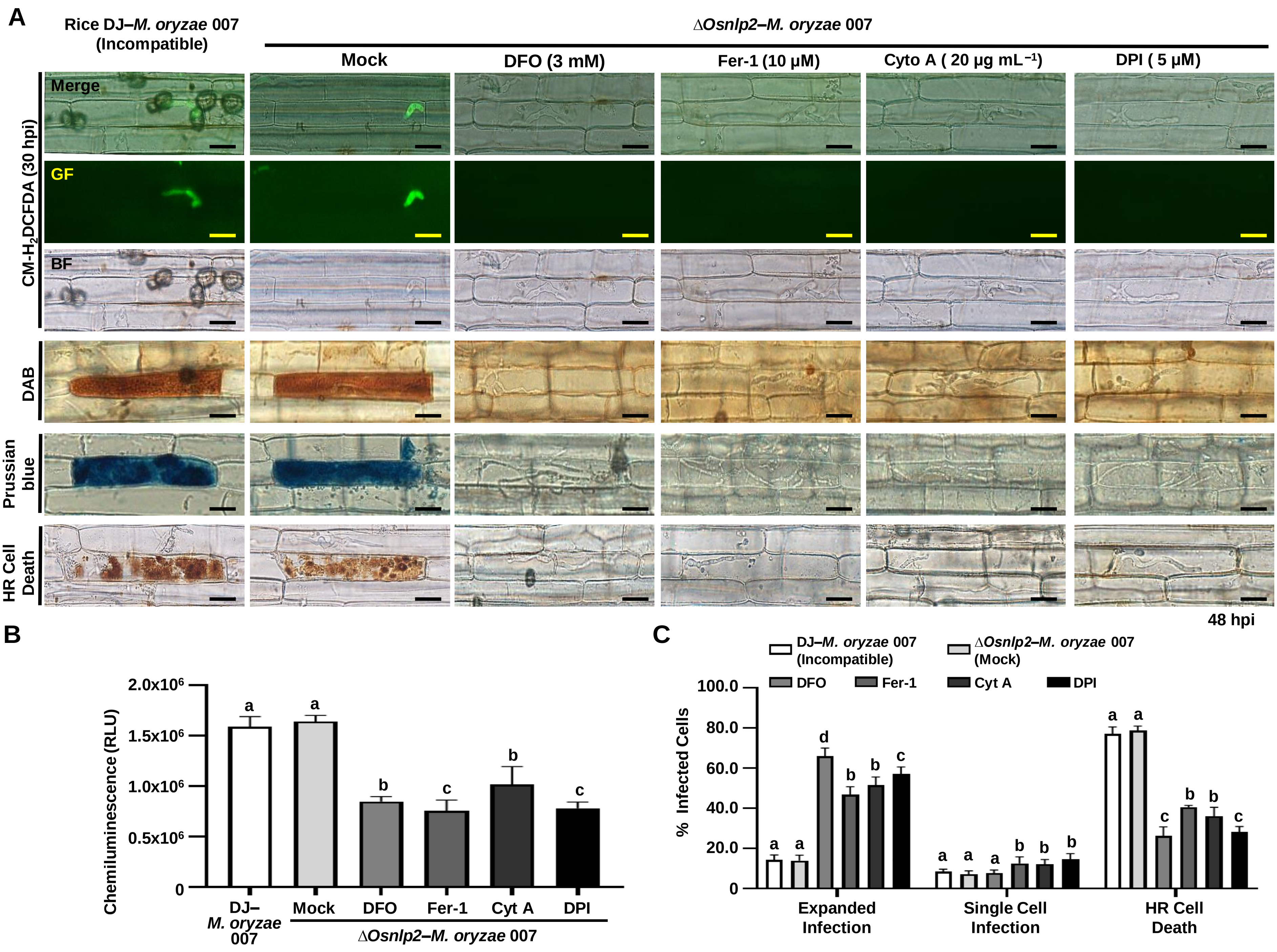
A
Rice DJ–M. oryzae 007 (Incompatible)
ΔOsnlp2–M. oryzae 007
Mock
DFO (3 mM)
Fer-1 (10 μM)
Cyto A (20 μg mL⁻¹)
DPI (5 μM)
CM-H2DCFDA (30 hpi)
Merge
GF
BF
DAB
Prussian blue
HR Cell Death
48 hpi
B
Chemiluminescence (RLU)
2.0x10⁶
1.5x10⁶
1.0x10⁶
0.5x10⁶
0
a
a
b
c
b
c
DJ–M. oryzae 007
Mock
DFO
Fer-1
Cyt A
DPI
ΔOsnlp2–M. oryzae 007
C
DJ–M. oryzae 007 (Incompatible)
ΔOsnlp2–M. oryzae 007 (Mock)
DFO
Fer-1
Cyt A
DPI
% Infected Cells
100.0
80.0
60.0
40.0
20.0
0
a a d b b c
a a a b b b
a a c b b c
Expanded Infection
Single Cell Infection
HR Cell Death

Figure S9. Suppression of HR cell death in *ΔOsnlp2* leaf sheaths infected with avirulent *Magnaporthe oryzae* 007 after treatment with deferoxamine (DFO), ferrostatin-1 (Fer-1), cytochalasin A (Cyt A), and diphenyleneiodonium (DPI). Leaf sheaths of rice DJ and *ΔOsnlp2* mutant line # 4-2 were inoculated with *M. oryzae* 007. (A) Images of ROS and ferric ion accumulation in infected rice cells after treatment with ferroptosis suppressors. ROS accumulation was detected by CM-H_2DCFDA and DAB staining. Ferric ion (Fe^{3+}) accumulation was visualized by Prussian blue staining. The images shown were taken using a fluorescence microscope (Zeiss equipped with Axioplan 2). BF, bright field; GF, green fluorescence; hpi, hours post inoculation. Scale bars=10 μm. (B) ROS quantification in infected rice cells after treatment with ferroptosis suppressors. ROS quantities in rice cells were determined by a chemiluminescence assay. Values are means ± SD of relative luminescence units (RLU) (*n*=10) from different rice sheath discs. (C) Quantification of infection types in rice sheaths treated with ferroptosis suppressors. The cell numbers of different infection types were counted at 48 hpi using a microscope. The percentages of infected cells are presented as means ± SD from the cell numbers of infection types in rice sheaths of different plants (*n*=4). Different letters above the bars indicate significantly different means as determined by the least significant difference (LSD) test ($P<0.05$).

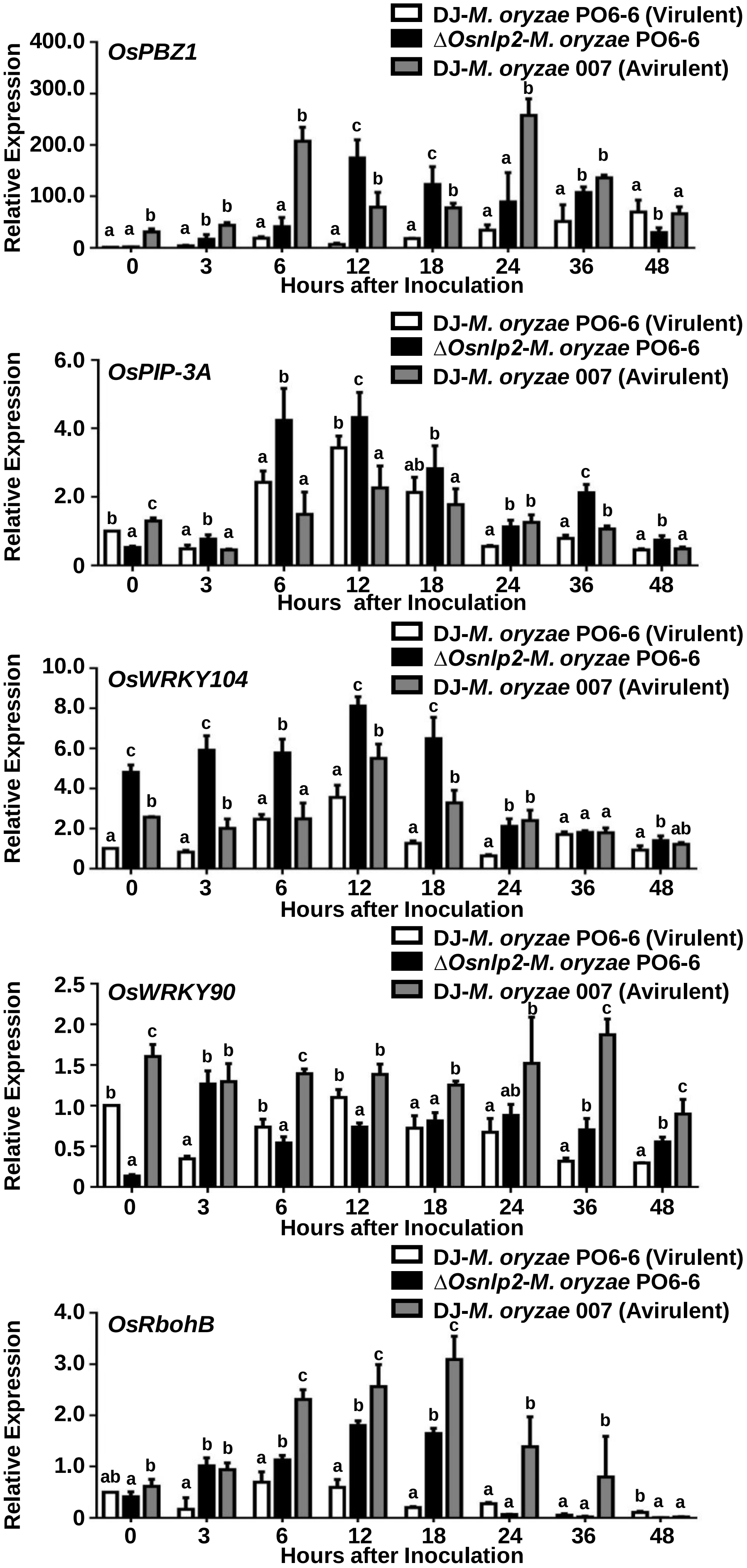

DJ-M. oryzae PO6-6 (Virulent)
ΔOsnlp2-M. oryzae PO6-6
DJ-M. oryzae 007 (Avirulent)
OsPBZ1
Relative Expression
400.0
300.0
200.0
100.0
0
0
3
6
12
18
24
36
48
Hours after Inoculation
OsPIP-3A
6.0
4.0
2.0
OsWRKY104
10.0
8.0
6.0
4.0
2.0
OsWRKY90
2.5
2.0
1.5
1.0
0.5
OsRbohB
4.0
3.0
2.0
1.0

Figure S10. Real-time qRT-PCR analysis of time-course expression of defense-related genes in leaf sheaths of rice DJ and *ΔOsnlp2* mutant plants infected with *Magnaporthe oryzae* PO6-6 (virulent) and 007 (avirulent). Relative expression levels of *OsPBZ1*, *OsPIP-3A*, *OsWRKY104*, *OsWRKY90*, and *OsRbohB* at different time points after inoculation were obtained by normalizing with respect to the expression levels of the internal control *OsUbiquitin*. The data are means ± SD of relative gene expression levels in rice leaf sheaths from three independent experiments. Different letters above the bars indicate significantly different means as determined by the least significant difference (LSD) test (P<0.05).
